# Sarcopenia and fat loss from serial CT predict survival in multiple myeloma patients undergoing stem cell transplantation

**DOI:** 10.1186/s12957-025-04007-6

**Published:** 2025-09-17

**Authors:** Julian Kylies, Elias Brauneck, Matthias Priemel, Dominik Kylies, Katja Weisel, Leon-Gordian Leonhardt, Lennart Viezens

**Affiliations:** 1 Department of Trauma and Orthopedic Surgery, University Medical Center Hamburg-Eppendorf, Martinistraße 52, Hamburg, 20246 Germany; 2 III. Department of Medicine, University Medical Center Hamburg-Eppendorf, Hamburg, Germany; 3 Hamburg Center for Kidney Health (HCKH), University Medical Center Hamburg-Eppendorf, Hamburg, Germany; 4 Department of Oncology, Hematology and Bone Marrow Transplantation With Section Pneumology, Hubertus Wald University Cancer Center Hamburg (UCCH), University Medical Center Hamburg-Eppendorf, Hamburg, Germany

## Abstract

**Background:**

Frailty and sarcopenia are associated with adverse outcomes in multiple myeloma (MM), but their longitudinal changes and clinical relevance remain unclear. This study evaluated the longitudinal changes in body composition parameters derived from computed tomography (CT) scans and their association with survival and functional decline in MM patients undergoing autologous stem cell transplantation (ASCT).

**Methods:**

We analyzed 49 MM patients who underwent three sequential CT scans between 2009 and 2024. CT-based body composition parameters—skeletal muscle index (SMI), paraspinal muscle index (PSMI), psoas muscle index (PMI), skeletal muscle density (SMD), and visceral adipose tissue (VAT)—were measured at the L3 level. Changes in these parameters were assessed over the disease course, and their impact on survival and functional outcome was evaluated using Cox proportional hazards regression models and Kaplan–Meier survival analyses.

**Results:**

CT morphometric body composition parameters declined significantly over time in both sexes. In males, SMI decreased from 48.6 ± 7.1 to 38.4 ± 8.1 cm^2^/m^2^ (–21%, *p* < 0.0001); in females, from 36.6 ± 7.8 to 26.9 ± 4.7 cm^2^/m^2^ (–26%, *p* < 0.0001). VAT declined from 115.9 ± 9.1 to 84.9 ± 9.7 cm^2^ in males (–27%, *p* < 0.0001) and from 63.5 ± 7.1 to 42.1 ± 7.7 cm^2^ in females (–34%, *p* < 0.0001). Patients < 55 years showed comparable declines (e.g., male SMI –21%, VAT –57%). High disease activity was associated with greater SMI (–31.1% vs. –15.5%, *p* < 0.001) and VAT (–33.5% vs. –26.5%, *p* < 0.01) losses versus low activity. ASCT patients had larger declines (SMI –31.1% vs. –15.4%, *p* < 0.001; VAT –33.4% vs. –27.5%, *p* < 0.01). Cox regression identified reductions in SMI (HR 1.40, 95% CI 1.10–2.20, *p* = 0.012) and VAT (HR 1.90, 95% CI 1.40–2.90, *p* = 0.002) as independent predictors of reduced survival. Patients with SMI loss ≥ 10% or VAT loss ≥ 12% had significantly shorter survival (SMI: 80.2 vs. 110.2 months, *p* = 0.01; VAT: 84.3 vs. 109.4 months, *p* < 0.01) and greater functional decline, with ECOG worsening from 1 to 3 (*p* < 0.0001).

**Conclusion:**

Longitudinal changes in SMI and VAT were significant predictors of survival and functional decline in MM patients undergoing ASCT. Routine CT-based body composition assessments might serve as valuable tools for additional risk stratification and potential targeted interventions. These findings underscore the importance of integrating body composition analysis into clinical practice for improved risk stratification and potential implementation of early intervention strategies.

**Supplementary Information:**

The online version contains supplementary material available at 10.1186/s12957-025-04007-6.

## Introduction

Multiple myeloma (MM) is a common hematologic malignancy [1, 2], characterized by the clonal proliferation of malignant plasma cells in the bone marrow [3], predominantly affecting elderly individuals [4–6]. Despite advances in treatment, including novel agents and autologous stem cell transplantation (ASCT), which have significantly improved survival outcomes, there remains a clinical need for individualized treatment strategies and reliable risk stratification tools—particularly for elderly patients [7–9]. In this population, treatment is often challenging due to comorbidities, physiological decline, and age-related changes in physical function, necessitating personalized approaches [10–12]. Frailty, characterized by reduced physiological reserve and increased vulnerability to stressors, has been shown to be associated with increased morbidity, treatment intolerance, and higher mortality in various cancer entities, including MM [13–15]. Frailty is commonly associated with aging and chronic illness but can also result from disease-related declines and the cumulative effects of intensive treatments like chemotherapy and ASCT [16]. Sarcopenia, characterized by the progressive loss of skeletal muscle mass and function, as well as by reductions in adipose tissue, is a frequent manifestation of frailty and contributes to increased morbidity and mortality [17, 18]. Traditional frailty assessments based on clinical scoring systems (e.g. Fried Frailty Phenotype [19], and the International Myeloma Working Group (IMWG) Frailty Score [20]) can be subjective, time-consuming, especially in routine practice and may not capture the full extent of body composition changes that accompany the disease course and aging.

In contrast, CT-morphometric assessments may offer a more objective and efficient alternative for evaluating sarcopenia and fat loss as correlates of clinical frailty [21]. CT morphometric parameters such as skeletal muscle index (SMI), paraspinal muscle index (PSMI), and psoas muscle index (PMI) are commonly used to assess muscle quantity [22], while skeletal muscle density (SMD) reflects muscle quality and fat infiltration [23]. In addition, visceral adipose tissue (VAT) serves as a surrogate for metabolic health and has been shown to affect survival in cancer patients [24].

While in some populations, the implications on clinical outcomes and survival have been established for CT-based body composition parameters, as demonstrated for upper gastrointestinal cancer, colon cancer and hepatocellular carcinoma [24–27], the interpretation in MM, to this day remains challenging [28–30]. One factor is, that MM reflects a highly heterogenous disease with treatment options ranging from watch and wait to stem cell transplantation depending on the patient status as well as on the clinical and molecular disease phenotype [31]. Another aspect is that the majority of CT-morphometric studies were cross-sectional, only incorporating a morphometric assessment at a single time point, thereby only providing limited insight into the trajectory of body composition over the longitudinal course of this complex disease [28–30].

To bridge this gap in knowledge we here analyzed the longitudinal changes in CT morphometric body composition parameters at three sequential time points and their implications on function and survival in patients with MM undergoing ASCT, a high-risk collective receiving aggressive treatment. Given the relatively small sample size and the complexity of longitudinal data acquisition, this study was designed as an exploratory observational analysis. Our findings aim to clarify the clinical importance of changes in body composition over time, with the goal of improving risk assessment and early intervention, and ultimately supporting more personalized treatment and care for high-risk patients with MM.

## Materials and methods

This study was approved by the local ethics committee (ID: 2025–300576-WF) and conducted in accordance with the Declaration of Helsinki. Due to its retrospective and anonymous design, the requirement for informed consent was waived. Patients were eligible for inclusion if they had a confirmed diagnosis of MM, underwent ASCT, and had three whole-body CT scans acquired after therapy initiation and performed at defined stages of the disease course between 2009 and 2024. Inclusion further required availability of corresponding clinical and laboratory data at the time of each CT scan. Patients were excluded if any of the CT scans were performed externally or did not follow institutional imaging protocols, CT image quality was insufficient for morphometric analysis (e.g., severe motion artifacts or incomplete abdominal coverage), complete clinical or laboratory data for at least one CT time point were missing, or other active malignancies or comorbid conditions (e.g., neuromuscular disorders, chronic inflammatory diseases, or cachexia of non-myeloma origin) were present that could independently affect skeletal muscle mass and confound sarcopenia-related measurements. 49 Patients met this criteria and were included in the study. All CT scans were conducted after therapy initiation. The three CT scans served as reference points for data evaluation (tCT1, tCT2, tCT3). In all patients, CT imaging was performed at clinically standardized milestones: after completion of last cycle of induction chemotherapy (tCT1), following consolidation or early maintenance therapy (tCT2), and approximately one year later during routine long-term follow-up (tCT3). Clinical assessments and laboratory tests were performed on the same day as each CT scan, ensuring temporal alignment between imaging-based body composition measurements and clinical/laboratory parameters. All imaging was performed at our institution using the same CT systems and clinical protocols, ensuring standardized acquisition parameters and enhancing comparability, particularly for HU measurements.

### Treatment protocols and study endpoint

All patients received standard-of-care treatment in accordance with the German S3 guidelines for MM, as recommended by the German Society for Hematology and Medical Oncology (DGHO) and the German Working Group for Blood and Marrow Transplantation (DAG-KBT) [32]. First-line induction therapy consisted of a combination regimen including a proteasome inhibitor (e.g., bortezomib), an immunomodulatory agent (e.g., lenalidomide or thalidomide), and dexamethasone. In selected cases, a CD38-targeting monoclonal antibody such as daratumumab was added. Stem cell mobilization was performed using granulocyte colony-stimulating factor (G-CSF), with or without cyclophosphamide or plerixafor. Conditioning prior to ASCT was conducted with melphalan. Post-transplant maintenance therapy, typically with lenalidomide, with or without bortezomib, was administered based on cytogenetic risk profile and individual tolerability.

The primary endpoint of the study was overall survival, defined as the time from the first CT scan (tCT1) to death from any cause or last follow-up. Secondary endpoints included longitudinal changes in CT-morphometric parameters (SMI, PSMI, PMI, SMD, VAT), changes in functional performance status (ECOG), and analgesic use categorized according to the WHO analgesic ladder.

**Fig. 1:**
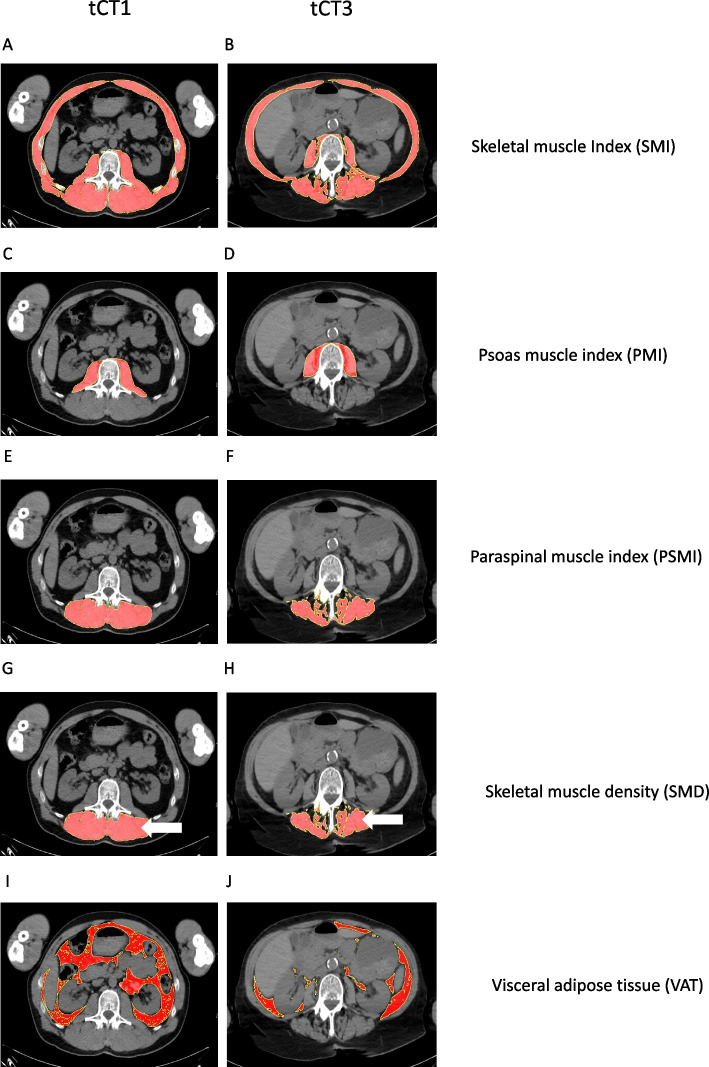
CT morphometric measurement of sarcopenia and visceral fat over the disease course. Shows example measurements of CT morphometric body composition parameters (Skeletal muscle Index (SMI) (**A** and **B**), Psoas muscle index (PMI) (**C** and **D**), Paraspinal muscle index (PSMI) (**E** and **F**), Skeletal muscle density (SMD) (**G** and **H**) (measured via the paraspinal muscles, indicated by arrows) and Visceral adipose tissue (VAT) (**I** and **J**) and its change over the disease course

CT-based morphometric parameters were measured using automated thresholding in combination with Fiji imaging software (Version 2.3.0/1.53q, Max Planck Institute of Molecular Cell Biology and Genetics), employing automated thresholding in combination with predefined Hounsfield Unit (HU) density ranges, as previously described [22, 33]. Figure 1 shows example CT scans and the associated measurements over the disease course (Fig. 1). Skeletal muscle was segmented using a standardized HU range from −29 to + 150, and VAT using a range from −190 to −30. These HU-based thresholds enabled selective segmentation of muscle and fat tissue, allowing for reproducible area measurements. After initial system calibration and definition of regions of interest (ROI) at the L3 level, the thresholding procedure was applied in batch mode across the full CT dataset. This workflow enabled automatic detection and quantification of cross-sectional muscle and fat areas, including SMI, PSMI, PMI, SMD, and VAT. Muscle areas were normalized to body height (cm^2^/m^2^), and SMD was determined by measuring the mean density (HU) of paraspinal muscle tissue.

Following automated batch segmentation, all measurements were visually reviewed for accuracy. Minor manual corrections were performed in cases of missegmentation due to artifacts, adjacent anatomical structures, or incomplete contrast between tissue compartments. When thresholding and segmentation were successful, fully automated measurement of one CT scan typically required approximately 30 s per scan. Laboratory values (serum creatinine, serum calcium, hemoglobin, serum and urinary M-protein) as well as clinical parameters (Visual Analogue Scale (VAS), analgesic use classified according to the World Health Organization (WHO) analgesic ladder (grades I–III), and functional status assessed using the Eastern Cooperative Oncology Group (ECOG) performance scale) were extracted from electronic patient records at the time of each respective CT scan (tCT1, tCT2, tCT3).

### Statistical analysis

Data analysis was performed using SPSS software (IBM SPSS Statistics v.29, IBM, Armonk, NY, USA). Graphical representation was performed using GraphPad Prism (v.10.2.2). Differences in CT morphometric frailty parameters, pain severity, analgesic use, and ECOG status across time points were evaluated using the non-parametric Friedman test, followed by post hoc pairwise comparisons with Bonferroni correction. Data are presented as mean ± standard deviation.

A Cox proportional hazards regression model was used to assess the influence of changes of tC1 to tC3 in SMI, VAT, SMD, PSMI, and PMI on survival, adjusting for the following covariates: age, sex, ECOG performance status, International Staging System (ISS) stage, cytogenetic risk status, and time from diagnosis to ASCT. The Cox regression model was fitted using partial likelihood estimation and the Efron method for handling ties. Hazard ratios (HRs) with 95% confidence intervals (CIs) were reported for all variables.

Furthermore, to determine cut-off values for changes in SMI and VAT that are predictive of survival outcomes, we conducted a Receiver Operating Characteristic (ROC) curve analysis. SMI and VAT SMD measurements were obtained from the first (tCT1) and last (tCT3) analysis time point. The percentage change in these parameters between these two time points was calculated for each patient. The optimal cut-off value for these parameters was identified using Youden’s Index (J). Subsequent survival analyses were conducted using Kaplan–Meier curves. Differences between curves were assessed using Log-Rank (Mantel-Cox) test. Statistical significance was set at *p* < 0.05.

## Results

### Basic demographic data and study design

In this study, 49 patients with Multiple Myeloma (MM) (22 female) undergoing ASCT were included. The basic demographic data are presented in Table 1. ECOG performance status at baseline (tCT1) was available for all patients. In total, 4 patients (11.8%) had ECOG 0, 15 patients (44.1%) had ECOG 1, 9 patients (26.5%) had ECOG 2, and 6 patients (17.6%) had ECOG 3. No patient had ECOG 4. ISS stage was determined at diagnosis in all patients. 15 patients (31%) had ISS Stage I, 16 patients (33%) had Stage II, and 18 patients (36%) had Stage III, reflecting a distribution across standard and advanced disease (Table 1). The mean interval between each CT scan was 17.2 ± 3.4 months, with a mean total follow-up duration of 36.5 ± 6.8 months for CT evaluations (tCT1 to tCT3). Given that absolute frailty parameter values differ between sexes—since men typically have higher muscle mass—frailty parameters were analyzed separately for male and female patients (Suppl. Table 1). However, for percentage changes in these parameters from tCT1 to tCT3, both genders were analyzed together, as relative values provide a standardized comparison.

**Table 1:** Patient characteristics at baseline

Total (n)	49
Gender	N
Female	22
Male	27
Age	Mean (SD)
Total	67.1 (11.2)
Female	66.5 (9.8)
Male	67.9 (8.9)
BMI (kg/m^2^)	23.8
Cytogenetic risk	N (%)
Low	22 (45%)
High^*^	20 (41%)
Unknown	7 (14%)
IgG Type	29 (59%)
ISS	N (%)
I	15 (31%)
II	16 (33%)
III	18 (36%)
ECOG score at baseline	Median (Range)
Female	1.0 (0.9)
Male	1.0 (0.9)
	N (%)
ECOG Score 0	6 (12%)
ECOG Score 1	22 (45%)
ECOG Score 2	13 (27%)
ECOG Score 3	8 (16%)
ECOG Score 4	0 (0%)

Shows the baseline characteristics of the patient cohort. *Abbreviations* *BMI* Body Mass Index, *ISS* International Staging System

^*^The definition of high risk cytogenetics refers to "Revised International Staging System for Multiple Myeloma: A Report From International Myeloma Working Group" [34]

### Significant decline in CT morphometric analysis of sarcopenia and visceral fat

**Fig. 2:**
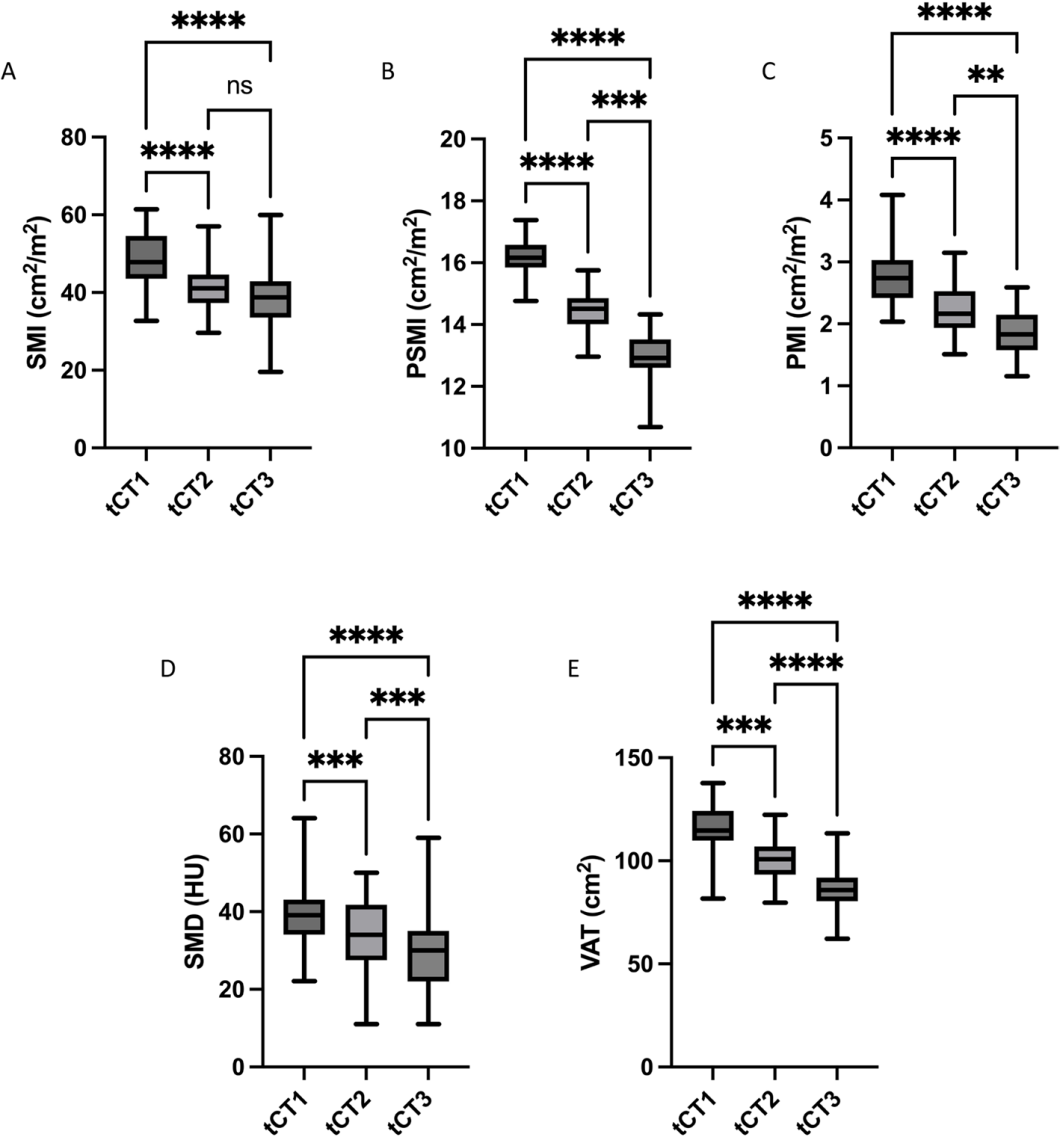
CT morphometric decline in muscle-associated parameters and visceral fat in male Patients. CT morphometric parameters in the male cohort showed a significant decline over the disease course from tCT1 to tCT3, indicating progressive muscle loss and fat reduction. Skeletal muscle index (SMI) decreased significantly from tCT1 to tCT2 and further to tCT3 (*p* < 0.0001) (**A**), with the decline between tCT2 and tCT3 approaching significance. Paraspinal muscle index (PSMI) showed a continuous and significant reduction across all time points (*p* < 0.0001) (**B**). Psoas muscle index (PMI) (**C**) decreased significantly between each time point (*p* < 0.0001). Skeletal muscle density (SMD) (**D**), which reflects muscle quality, declined significantly throughout the disease course (*p* < 0.001). Visceral adipose tissue (VAT) (**E**) also exhibited a progressive and significant reduction at each time point (*p* < 0.001), suggesting substantial disease-driven fat loss

In the male cohort, all CT morphometric parameters (SMI, PSMI, PMI, SMD, VAT) showed a significant decline over the disease course (reported as mean ± SD). SMI, an indicator of overall muscle size, decreased significantly from tCT1 (48.6 ± 7.1 cm^2^/m^2^) to tCT2 (40.6 ± 6.0 cm^2^/m^2^; *p* < 0.0001) and further to tCT3 (38.4 ± 8.1 cm^2^/m^2^; *p* < 0.0001 for tCT1 vs. tCT3). Although there was a decline between tCT2 and tCT3, this difference did not reach statistical significance (*p* = 0.12) (Fig. 2A). PSMI also demonstrated a significant reduction, starting from tCT1 (16.7 ± 0.5 cm^2^/m^2^) to tCT2 (14.9 ± 0.8 cm^2^/m^2^; *p* < 0.0001) and further to tCT3 (12.4 ± 0.7 cm^2^/m^2^; *p* < 0.0001 for tCT1 vs. tCT3). The decrease from tCT2 to tCT3 remained significant (*p* < 0.001) (Fig. 2B). Similarly, PMI decreased significantly at each time point, with values dropping from tCT1 (2.8 ± 0.6 cm^2^/m^2^) to tCT2 (2.4 ± 0.4 cm^2^/m^2^; *p* < 0.0001) and reaching lowest values at tCT3 (1.8 ± 0.7 cm^2^/m^2^; *p* < 0.0001 for tCT1 vs. tCT3). Each comparison between time points showed significant differences (*p* < 0.01) (Fig. 2C). SMD, reflecting muscle quality, declined significantly from tCT1 (40.1 ± 8.2 HU) to tCT2 (34.8 ± 8.0 HU; *p* < 0.001) and further to tCT3 (31.9 ± 10.0 HU; *p* < 0.001 for tCT1 vs. tCT3) (Fig. 2D). VAT exhibited a progressive and significant decrease as well, starting at tCT1 (115.9 ± 9.1 cm^2^) and dropping to tCT2 (100.0 ± 9.1 cm^2^; *p* < 0.001) and tCT3 (84.9 ± 9.7 cm^2^; *p* < 0.0001 for tCT1 vs. tCT3) (Fig. 2E). The reduction between each time point was statistically significant (*p* < 0.001).

**Fig. 3:**
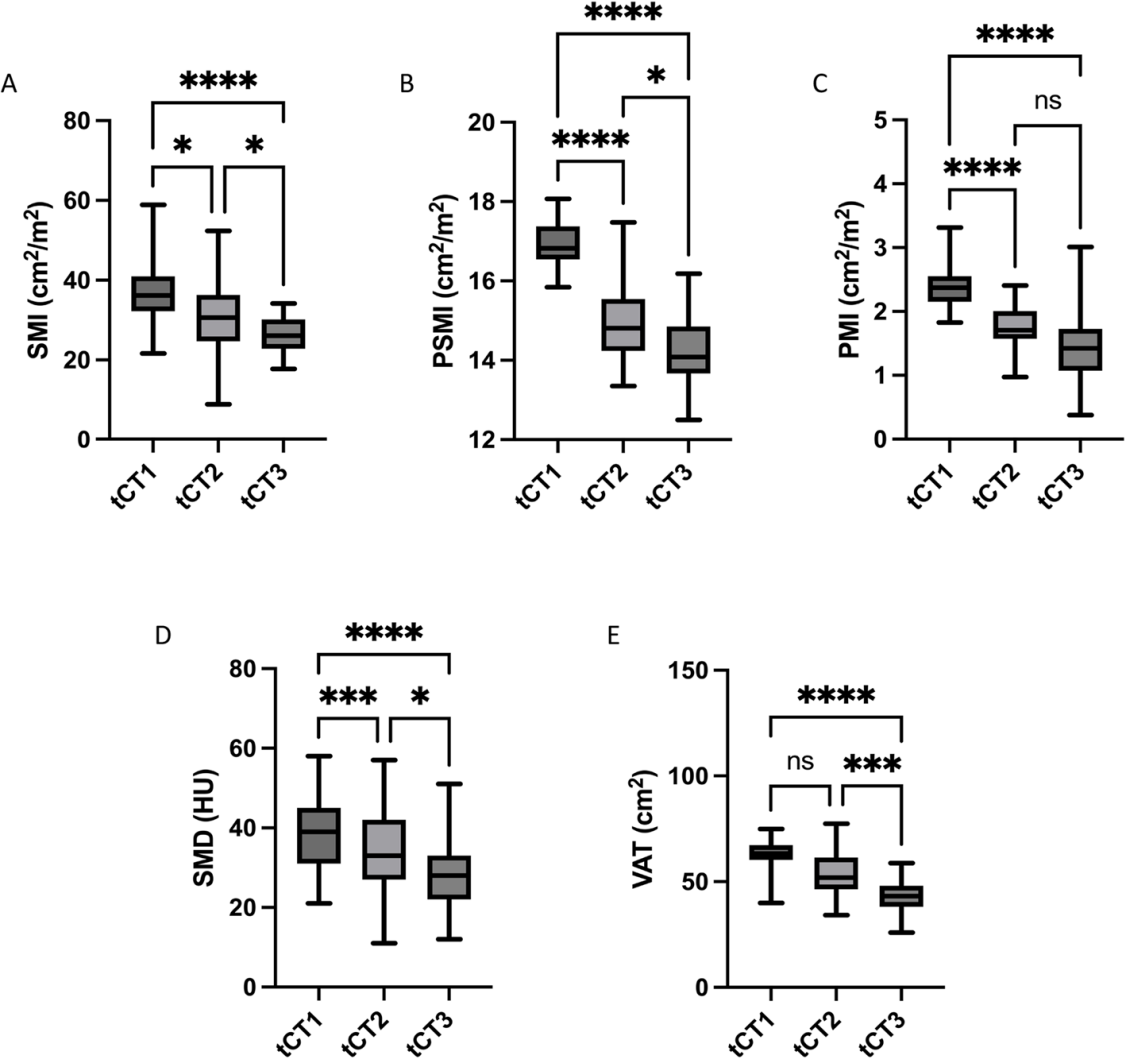
CT morphometric decline in muscle-associated parameters and visceral fat in female Patients. CT morphometric parameters in the female cohort followed a similar pattern as the male cohort, with significant reductions over time in muscle mass, muscle quality, and fat. SMI decreased significantly between tCT1 and tCT3 (*p* < 0.0001), with a significant decline also observed between tCT2 and tCT3 (*p* < 0.01). PSMI decreased continuously across all time points (*p* < 0.0001). PMI declined significantly between tCT1 and tCT2 (*p* < 0.0001), with no significant change observed between tCT2 and tCT3. SMD showed a marked reduction over time, with significant differences between tCT1 and tCT2 and between tCT1 and tCT3 (*p* < 0.001). VAT significantly declined from tCT1 to tCT3 (*p* < 0.0001), although the decline between tCT1 and tCT2 did not reach statistical significance

In the female cohort, all CT morphometric parameters (SMI, PSMI, PMI, SMD, VAT) significantly declined over the disease course, similarly to the male cohort (Fig. 3). SMI decreased significantly from tCT1 (36.6 ± 7.8 cm^2^/m^2^) to tCT2 (30.5 ± 8.0 cm^2^/m^2^; *p* = 0.02) and further to tCT3 (26.9 ± 4.7 cm^2^/m^2^; *p* < 0.0001 for tCT1 vs. tCT3). The decline between tCT2 and tCT3 was also significant (*p* = 0.01) (Fig. 3A). PSMI showed a steady and significant reduction across all time points, from tCT1 (16.9 ± 0.9 cm^2^/m^2^) to tCT2 (14.1 ± 0.6 cm^2^/m^2^; *p* < 0.0001) and further to tCT3 (13.9 ± 0.8 cm^2^/m^2^; *p* < 0.0001 for tCT1 vs. tCT3) (Fig. 3B). PMI declined significantly from tCT1 (2.5 ± 0.3 cm^2^/m^2^) to tCT2 (1.6 ± 0.4 cm^2^/m^2^; *p* < 0.0001), but the difference between tCT2 and tCT3, although reduced, did not reach statistical significance (*p* = 0.09) (Fig. 3C). SMD decreased markedly throughout the disease course, with significant reductions from tCT1 (38.7 ± 9.1 HU) to tCT2 (34.3 ± 10.1 HU; *p* < 0.001) and from tCT2 to tCT3 (28.7 ± 8.2 HU; *p* = 0.01) (Fig. 3D). VAT showed a reduction over the disease course as well, decreasing from tCT1 (63.5 ± 7.1 cm^2^) to tCT3 (42.1 ± 7.7 cm^2^; *p* < 0.0001). While there was decline from tCT1 to tCT2 (52.9 ± 9.1 cm^2^) as well, this difference was not statistically significant (*p* = 0.08) (Fig. 3E).

To better understand the potential impact of ASCT on longitudinal body composition changes, we performed a comparative analysis between patients with MM who underwent ASCT (*n* = 49) and a separate cohort of patients with MM who did not undergo ASCT (*n* = 47). The non-ASCT group met the same inclusion and exclusion criteria—except for not receiving ASCT—and had also undergone three whole-body CT scans during routine clinical follow-up, acquired at comparable stages of the disease course. Their imaging data were not included in the main ASCT cohort analysis but served as an independent comparison group to assess the influence of intensive treatment on CT morphometric changes. From tCT1 to tCT3 (reported as mean ± SD), SMI decreased by 31.1 ± 13.4% in ASCT patients, nearly twice the reduction observed in the non-ASCT group (15.4 ± 14.6%, *p* < 0.001; Supplementary Figure 1 A). Similarly, PSMI declined more profoundly in ASCT patients (29.1 ± 6.5%) than in non-ASCT patients (18.1 ± 5.3%, *p* < 0.001; Supplementary Figure 1B). PMI showed the most pronounced reduction, with a mean decrease of 40.6 ± 13.1% in the ASCT group versus 25.8 ± 13.7% in the non-ASCT group (*p* < 0.001; Supplementary Figure 1 C). In contrast, SMD declined similarly in both groups (27.5 ± 18.9% in ASCT vs. 24.7 ± 21.5% in non-ASCT patients, *p* = 0.22; Supplementary Figure 1D). VAT also declined significantly in both groups, but more markedly in ASCT patients (33.4 ± 9.2%) compared to non-ASCT patients (27.5 ± 14.7%, *p* < 0.01; Supplementary Figure 1E).

In both the male and female cohorts, all CT morphometric parameters (SMI, PSMI, PMI, SMD, VAT) significantly declined over the disease course, reflecting a progressive loss of muscle mass, muscle quality, and visceral adipose tissue. Notably, patients with MM undergoing ASCT exhibited significantly greater reductions in these parameters compared to non-ASCT patients, indicating a more pronounced deterioration in body composition.

### Subgroup analysis by age: CT morphometric decline in patients under 55 years suggests disease-driven effects on body composition

To assess whether the observed deterioration in body composition was primarily disease-related rather than a result of age-associated sarcopenia, we performed a dedicated subgroup analysis of patients younger than 55 years. Since physiological aging processes are less likely to account for frailty in this population, any significant decline in CT morphometric parameters may reflect myeloma-related or treatment-induced mechanisms. We therefore analyzed longitudinal changes in muscle mass, muscle quality, and visceral adiposity separately for male and female patients in this younger cohort.

This subgroup included 15 patients (9 female) with a mean age of 51.2 ± 3.3 years. Significant declines were observed in nearly all CT morphometric parameters over the course of the disease (tCT1 to tCT3) (reported in mean/SD). SMI declined from 48.5 ± 6.6 cm^2^/m^2^ at tCT1 to 38.1 ± 7.4 cm^2^/m^2^ at tCT2 (*p* = 0.02), and further to 34.5 ± 13.0 cm^2^/m^2^ at tCT3 (*p* < 0.001 for tCT1 vs. tCT3), while the difference between tCT2 and tCT3 was not statistically significant (Supplementary Figure 2 A). PSMI dropped significantly from 16.5 ± 0.8 cm^2^/m^2^ at tCT1 to 14.5 ± 0.8 cm^2^/m^2^ at tCT2 (*p* < 0.01), and to 13.5 ± 0.8 cm^2^/m^2^ at tCT3 (*p* < 0.0001 for tCT1 vs. tCT3); tCT2 vs. tCT3 was not significant (Supplementary Figure 2B). PMI declined from 2.6 ± 0.3 cm^2^/m^2^ at tCT1 to 2.1 ± 0.4 cm^2^/m^2^ at tCT2 (*p* < 0.01), and 1.9 ± 0.5 cm^2^/m^2^ at tCT3 (*p* < 0.001 for tCT1 vs. tCT3), with no significant difference between tCT2 and tCT3 (Supplementary Figure 2 C). SMD values dropped from 40.3 ± 9.7 HU at tCT1 to 35.9 ± 10.9 HU at tCT2 and 32.6 ± 11.7 HU at tCT3; the decline was only significant between tCT1 and tCT3 (*p* < 0.01) (Supplementary Figure 2D). VAT decreased significantly from 104.2 ± 24.5 cm^2^ at tCT1 to 83.4 ± 20.6 cm^2^ at tCT2 (*p* = 0.03), and further to 67.3 ± 27.1 cm^2^ at tCT3 (*p* < 0.0001 for tCT1 vs. tCT3, and *p* = 0.02 for tCT2 vs. tCT3) (Supplementary Figure 2E).

To determine whether younger patients exhibited a distinct pattern of body composition decline compared to older patients, we further compared the relative percentage changes in CT morphometric parameters between patients aged < 55 years and those aged ≥ 55 years from tCT1 to tCT3 (Supplementary Figure 3) (reported as mean/SD). While both subgroups demonstrated significant declines in skeletal muscle mass, muscle quality, and visceral adipose tissue over the course of the disease, there were no statistically significant differences in the magnitude of these changes between age groups. Specifically, SMI declined by − 28.9 ± 25.9% in patients < 55 years and − 21.7 ± 18.2% in those ≥ 55 years (*p* = n.s.) (Supplementary Figure 3 A). Similarly, changes in PSMI (− 18.3 ± 5.0% vs. − 18.4 ± 5.7%, *p* = n.s.) (Supplementary Figure 3B), PMI (− 27.8 ± 17.7% vs. − 34.0 ± 22.2%, *p* = n.s.) (Supplementary Figure 3 C), SMD (− 19.0 ± 24.1% vs. − 26.6 ± 17.5%, p = n.s.) (Supplementary Figure 3D), and VAT (− 34.0 ± 14.4% vs. − 27.9 ± 12.3%, *p* = n.s.) (Supplementary Figure 3E) were comparable between groups.

Taken together, these findings suggest that both younger and older patients with MM are similarly vulnerable to disease- or treatment-associated body composition decline, supporting the hypothesis that sarcopenia and adipose tissue loss in MM are driven by factors beyond chronological aging.

### Subgroup analysis by disease activity: high disease activity accelerates loss of skeletal muscle and visceral adipose tissue

To evaluate the impact of disease activity (DA) on frailty parameters throughout the disease course, we compared changes in CT morphometric parameters between patients with high (22 patients) and low DA (27 patients). High DA was defined by the presence of one or more CRAB criteria and/or elevated M-protein levels (serum M-protein ≥ 3 g/dL or urine M-protein ≥ 500 mg/24 h), while patients without these features were classified as having low DA. The percentage change in CT morphometric parameters from tCT1 to tCT3 was analyzed for both groups.

**Fig. 4:**
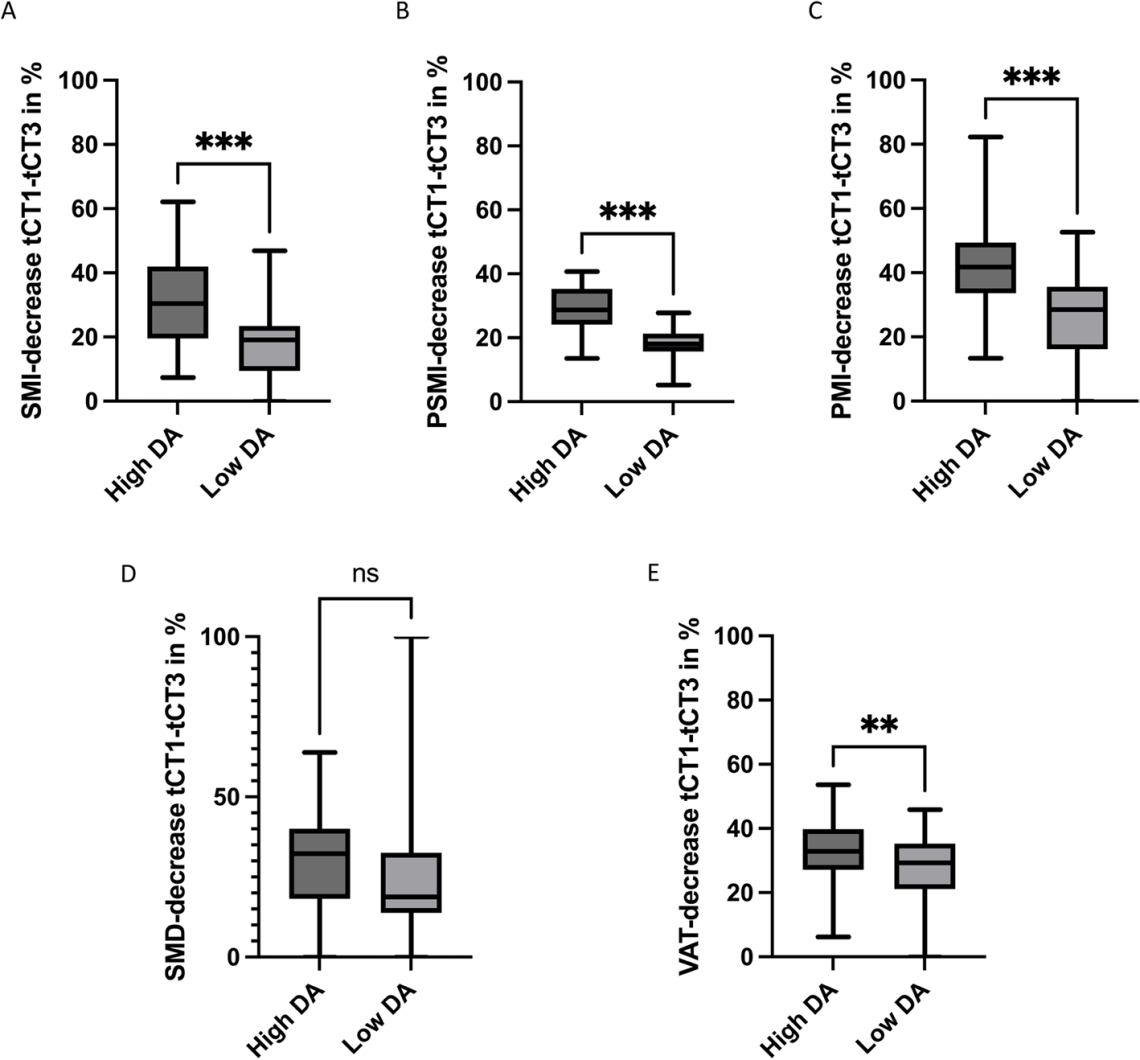
Impact of disease activity on CT morphometric sarcopenia and visceral body fat. Patients with high disease activity (DA) experienced significantly greater declines in muscle mass and fat compared to those with low DA, reflecting accelerated frailty progression. SMI, PSMI, and PMI all showed significantly larger reductions in the high DA group (*p* < 0.001). VAT also declined more substantially in high DA patients compared to low DA patients (*p* < 0.01). In contrast, SMD remained relatively stable, with no significant difference between the high and low DA groups

The results showed that DA had a significant impact on SMI, PSMI, PMI, and VAT (Fig. 4). Patients with high DA exhibited more pronounced declines compared to those with low DA (reported as mean ± SD). SMI decreased by 31.1% ± 13.5% in the high DA group, compared to 15.5% ± 14.5% in the low DA group (*p* < 0.001) (Fig. 4A). PSMI followed a similar trend, with a mean decline of 29.1% ± 6.5% in the high DA group versus 18.1% ± 5.2% in the low DA group (*p* < 0.001) (Fig. 4B). PMI also showed a significantly greater reduction in the high DA group (41.9% ± 13.4%) compared to the low DA group (25.8% ± 13.7%; *p* < 0.001) (Fig. 4C). SMD remained relatively stable across both groups, with no significant difference between high DA (27.5% ± 18.9%) and low DA (24.7% ± 21.5%; *p* = 0.44) (Fig. 4D), indicating that muscle quality was not substantially affected by DA. VAT showed a substantial difference between the groups as well. Patients with high DA experienced a mean VAT reduction of 33.5% ± 10.0%, compared to 26.5% ± 13.8% in the low DA group (*p* < 0.01) (Fig. 4E).

These findings highlight the impact of DA on body composition in multiple myeloma patients, with high DA contributing to accelerated frailty, reflected by substantial reductions in SMI, PSMI, PMI, and VAT over time.

### Significant decreases in SMI and VAT are independent predictors of poor survival and functional decline in multiple myeloma patients undergoing ASCT

To assess the clinical implications of CT morphometric changes, we analyzed the relationship between frailty parameters and survival outcomes in patients with MM undergoing ASCT. The survival analysis aimed to determine whether changes in skeletal muscle mass, VAT, and other morphometric and clinical parameters could serve as independent predictors of overall survival.

Analysis of CT-morphometric frailty parameters using a Cox proportional hazards regression model identified t1-t3-changes in SMI and VAT as independent predictors of decreased survival in patients with MM undergoing ASCT (Table 2). After adjusting for age, sex, ECOG performance status, ISS stage, cytogenetic risk status, and time from diagnosis to ASCT, a decrease in SMI was associated with a significantly increased risk of death (HR = 1.40, 95% CI: 1.10–2.20, *p* = 0.012), indicating that patients with a greater loss of skeletal muscle mass had poorer survival. Similarly, VAT-change was a strong and independent predictor of survival, with a decrease in VAT associated with a markedly increased risk of death (HR = 1.90 95% CI: 1.40–2.90, *p* = 0.002). Interestingly, while high-risk cytogenetics; HR = 2.35, 95% CI: 1.15–4.80, *p* = 0.021), baseline ECOG performance status (HR = 1.45, 95% CI: 1.05–1.98, *p* = 0.03), and ISS stage (HR = 1.85, 95% CI: 1.02–3.10, *p* = 0.04) were significant predictors of survival (Table 2), the most striking finding was that both SMI and VAT changes from tCT1 to tCT3 emerged as independent risk factors for mortality. ECOG performance status was treated as an ordinal variable (0 to 4), and ISS stage as categorical (Stage I–III). Both parameters were included as covariates to adjust for functional and disease-related risk at baseline.

**Table 2:** Uni- and multivariable Cox regression models for overall survival (OS)

Variable	HR (univariable	95% CI (univariable)	*p*-Value (univariable)	HR (multivariable)	95% CI (mulrivariable	*p*-value (multifariable)
*SMI-change	1.50	1.20–2.10	0.005	1.40	1.10–2.20	0.012
*VAT-change	2.10	2.00–4.10	0.001	1.90	1.40–2.90	0.002
PSMI-change	1.80	0.40–8.00	0.25	6.12	0.50–74.80	0.38
PMI-change	1.50	0.60–3.50	0.18	1.75	0.70–4.30	0.29
SMD-change	0.50	0.20–1.20	0.10	0.45	0.18–1.12	0.18
Age	1.01	0.99–1.03	0.10	1.02	0.98–1.05	0.15
Time to ASCT	1.10	0.95–1.25	0.12	1.12	0.91–1.35	0.21
Sex	1.75	0.90–3.50	0.07	1.88	0.93–3.82	0.08
*High-risk cytogenetics	2.10	1.20–3.80	0.008	2.35	1.15–4.80	0.021
*ECOG base-line	1.50	1.10–3.20	0.02	1.45	1.05–1.98	0.03
*ISS baseline	1.90	1.10–3.20	0.03	1.85	1.02–3.10	0.04

Shows CT-morphometric and clinical parameters and the changes over the disease course

*significance in any way

*Abbreviations* *ECOG* Eastern Cooperative Oncology Group, *VAS* Visual analogue scale, *WHO* World Health Organization pain ladder

High-risk cytogenetics were defined based on the mSMART 3.0 criteria in use during the study period, including del(17p), t(4;14), t(14;16), and t(14;20). We acknowledge that newer classifications such as mSMART 4.0 include additional markers like amp(1q) and del(1p), which were not consistently available in our cohort due to the retrospective nature and evolving diagnostic practices. Future studies should evaluate the interaction of these additional abnormalities with longitudinal body composition changes.

Our findings highlight the potential prognostic value of longitudinal body composition assessments in predicting survival outcomes beyond traditional clinical markers.

Other morphometric parameters, including PSMI-change (HR = 6.12, 95% CI: 0.50–74.80, *p* = 0.38), PMI-change (HR = 1.75, 95% CI: 0.70–4.30, *p* = 0.29), and SMD-change (HR = 0.45, 95% CI: 0.18–1.12, *p* = 0.18), were not statistically significant predictors of survival. Age (HR = 1.02, 95% CI: 0.98–1.05, *p* = 0.15), Sex (HR = 1.88, 95% CI: 0.93–3.82, *p* = 0.08) and time to ASCT (HR = 1.12, 95% CI: 0.91–1.35, p = 0.21) showed a trend toward increased risk but did not reach statistical significance (Table 2).

**Fig. 5:**
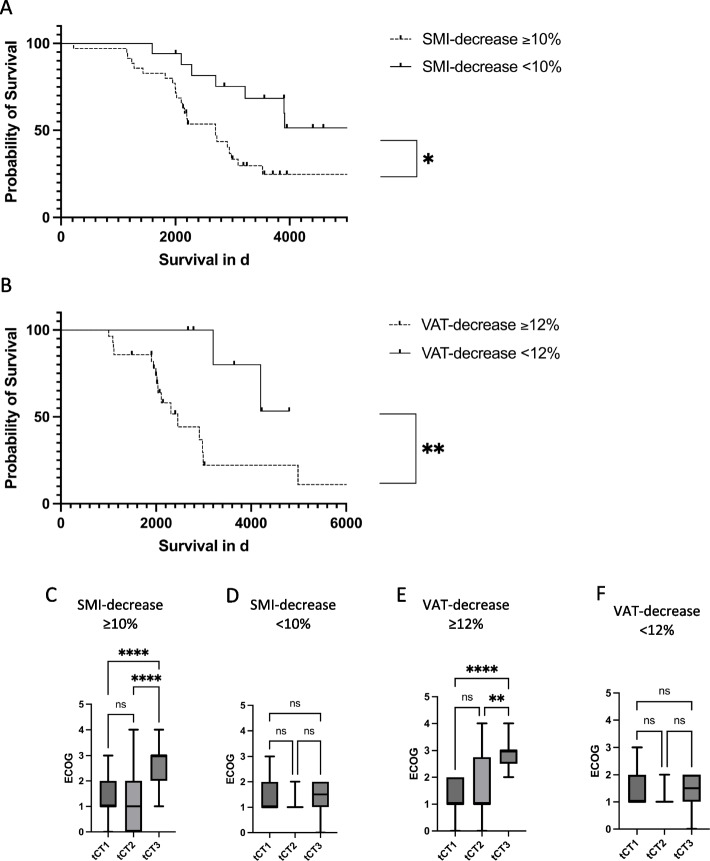
Survival and functional decline based on CT morphometric parameters. Kaplan–Meier survival analysis and ECOG performance status changes illustrate the clinical impact of morphometric changes on patient outcomes. Patients with a decrease in SMI of ≥ 10% or VAT of ≥ 12% had significantly reduced survival compared to those with lesser reductions (*p* < 0.01). Kaplan–Meier curves show that these thresholds were strongly associated with decreased survival. ECOG performance status worsened significantly in patients with severe reductions in SMI and VAT (*p* < 0.0001), indicating a progressive decline in functional status. In contrast, patients with less than 10% SMI reduction and less than 12% VAT reduction showed no significant changes in ECOG scores over the disease course

To further explore the clinical relevance of these findings, Kaplan–Meier survival analyses were performed. Thresholds for significant changes in VAT and SMI were determined using Youden’s J index from ROC curve analysis. Patients with a decrease in SMI of ≥ 10% and a decrease in VAT of ≥ 12% were compared to those with lesser reductions. Patients with a SMI decrease of ≥ 10% exhibited a significantly decreased survival rate (median survival: 80.2 months vs. 110.2 months, *p* = 0.01), as did patients with a VAT decrease of ≥ 12% (median survival: 84.3 months vs. 109.4 months, *p* < 0.01) (Fig. 5A and B).

These findings underscore the prognostic significance of CT-morphometric frailty parameters, particularly VAT and SMI changes, in the survival of multiple myeloma patients undergoing ASCT. The thresholds derived from ROC analysis provide clinically actionable metrics for identifying high-risk patients.

In addition to the survival analysis, changes in ECOG performance status were assessed over the disease course in patients stratified by CT-morphometric frailty parameters. Patients with a decrease in SMI of ≥ 10% and VAT decrease of ≥ 12% demonstrated a significant increase in ECOG scores (reported as median ± range) from tCT1 to tCT3 (SMI decrease ≥ 10%: median ECOG score: tCT1 = 1, tCT3 = 3, *p* < 0.0001, VAT decrease ≥ 12%: median ECOG score: tCT1 = 1, tCT3 = 3, *p* < 0.0001) (Fig. 5C and E), indicating a progressive decline in functional status. In contrast, patients with less than 10% decrease in SMI and less than 12% decrease in VAT showed no significant change in ECOG scores over the same time period (SMI decrease < 10% median ECOG score: tCT1 = 1, tCT3 = 1, *p* = 0.44, VAT decrease < 12% median ECOG score: tCT1 = 1, tCT3 = 1, *p* = 0.22) (Fig. 5D and F).

These findings suggest that severe reductions in both SMI and VAT are not only associated with decreased survival but are also linked to worsening functional status, as reflected by increasing ECOG scores over the disease course. This further highlights the potential clinical relevance of monitoring CT-morphometric frailty parameters to predict and potentially intervene in functional decline in multiple myeloma patients.

## Discussion

As an exploratory analysis, this study highlights longitudinal trends in CT-based body composition parameters among patients with MM undergoing ASCT. We demonstrate that muscle mass, muscle quality, and VAT decline over the disease course, with more pronounced changes observed in patients with high disease activity and in younger individuals. Importantly, reductions in SMI and VAT emerged as independent predictors of reduced overall survival and functional decline, underscoring their value as potential objective biomarkers for risk stratification.

Our findings confirm that CT-morphometric parameters decline over time in patients with MM, regardless of age. Patients with high disease activity—as defined by CRAB criteria and elevated M-protein levels—experienced more substantial losses in muscle and fat mass. Similarly, patients undergoing ASCT showed greater morphometric decline than those who did not, suggesting a possible treatment-related contribution. Notably, the ASCT group included patients with high-risk features such as SLiM criteria, high plasma cell infiltration, or adverse cytogenetics (e.g., del(17p), t(4;14)) even in the absence of CRAB criteria.

Despite other factors in patients with MM, the consistent reductions in muscle mass and VAT observed in both younger patients and those with high DA suggest these changes may be driven by disease- or treatment-related mechanisms as well. This raises the possibility that systemic inflammation and metabolic dysregulation could play a central role in driving these body composition changes in MM.

These observations are in line with emerging evidence that links progressive sarcopenia and fat loss in MM to a complex interplay of biological mechanisms, including chronic inflammation, cytokine dysregulation, and altered energy metabolism [35, 36]. Elevated levels of pro-inflammatory cytokines such as interleukin-6 (IL-6) and tumor necrosis factor-alpha (TNF-α) are commonly found in patients with MM and have been implicated in promoting both muscle catabolism and adipose tissue depletion. IL-6, produced by malignant plasma cells and the bone marrow microenvironment, can activate the JAK/STAT3 signaling pathway, leading to muscle protein breakdown via the ubiquitin–proteasome system [37]. TNF-α further exacerbates muscle wasting by stimulating NF-κB activation and impairing muscle regeneration [38, 39]. In parallel, inflammation-induced insulin resistance and altered lipid metabolism may drive visceral fat loss, as increased lipolysis and reduced adipogenesis have been observed in cancer-associated cachexia models [40]. Additionally, systemic metabolic shifts in MM—such as mitochondrial dysfunction, increased oxidative stress, and altered amino acid turnover—may contribute to the loss of lean and fat mass over time [41]. These biological processes not only promote physical decline but may also impair treatment response, aggravate fatigue, and reduce quality of life. Therefore, understanding the molecular underpinnings of sarcopenia and fat loss in MM is essential for identifying potential therapeutic targets and developing strategies to prevent or reverse disease-associated frailty. Furthermore, studies suggest that targeted strategies such as nutritional support, physical rehabilitation, and anti-inflammatory therapies may help mitigate these effects and improve outcomes in the future [42, 43]. Early identification of patients at risk through body composition monitoring could provide an opportunity for timely interventions, potentially slowing disease-related frailty progression and enhancing quality of life.

Secondly, we could demonstrate that decreases in CT-assessed sarcopenia and visceral body fat were associated with decreased overall survival and function in our select patient cohort. Previous research already demonstrated the prognostic significance of muscle mass and fat loss in cancer patients [24–27]. In MM, however, the data remained contradictory. While some studies have linked CT-defined sarcopenia at diagnosis to worse overall survival, others found no significant association [28–30]. Most of these studies relied on cross-sectional data, limiting the understanding of how body composition evolves over time and often focusing on single parameters. By adopting a longitudinal approach—to our knowledge the first of its kind—this study provides a more comprehensive perspective, demonstrating that changes in body composition, rather than single baseline assessments, are closely linked to poorer survival in select subgroups of high risk patients with MM. Notably, in our cohort, an SMI decrease of ≥ 10% and a VAT reduction of ≥ 12% were strong predictors of both reduced survival and functional decline, as indicated by worsening ECOG performance scores.

These findings suggest that CT-based body composition assessments over the disease course could serve as valuable tools for risk stratification in patients with MM undergoing ASCT. The identified thresholds for SMI and VAT reductions offer clinically actionable metrics to identify patients at risk for progressive sarcopenia and poor survival. Furthermore, these assessments may complement traditional frailty evaluation tools, offering a more objective and quantitative approach. Compared to clinical scoring systems, CT morphometric assessments are less subjective, reproducible, and integrate seamlessly into routine imaging workflows, making them a practical alternative in both research and clinical settings [19, 20].

Our study has several strengths and limitations worth noting. The strengths of this study include its longitudinal design, which enabled the evaluation of dynamic changes in body composition over time, and the comprehensive analysis of multiple CT-based parameters. To the best of our knowledge, this is the first study to evaluate multiple CT-based body composition assessments in sequential CT scans. By incorporating both muscle mass and adipose tissue metrics, we provided a more holistic view of body composition changes in patients with MM. Furthermore, the focus on patients with MM receiving ASCT while excluding other treatment strategies aids selecting a more homogenous collective. However, several limitations must be acknowledged. The retrospective nature of the study introduces the potential for selection bias, and the relatively small sample size may limit the generalizability of the findings. Although we adjusted for several confounding factors, such as age, sex, and disease stage, residual confounding cannot be excluded. Furthermore, exclusively analyzing patients with MM who underwent ASCT may limit the translatability of our findings to other MM patient groups. Additionally, the study was conducted at a single center, which may limit the external validity of the results.

Further research is needed to validate these findings in larger, multicenter cohorts and to explore their applicability across diverse patient populations. Prospective studies incorporating more detailed functional assessments, such as grip strength or gait speed, alongside CT-based evaluations, could provide a more comprehensive understanding of frailty-related body composition changes. Moreover, integrating systemic biomarkers of inflammation and metabolism with CT morphometric parameters could enhance their prognostic value and guide personalized treatment approaches.

In conclusion, longitudinal changes in body composition, particularly reductions in SMI and VAT over the course of the disease, are significant and independent predictors of survival and functional decline in patients with MM undergoing ASCT. These findings highlight the value of CT-based assessments for identifying patients at risk of progressive sarcopenia and death in order to guide early interventions. Integrating these evaluations into routine clinical practice may improve risk stratification and ultimately enhance patient outcomes.

## Supplementary Information

Supplementary Material 1: Supplementary Table 1. CT-morphometric and clinical parameters over the disease course. Supplementary Table 1 shows the values and significance levels of the Uni- and multivariable Cox regression models for overall survival. Abbreviations: SMI: Skeletal muscle index, VAT: Visceral adipose tissue, PSMI: Paraspinal muscle index, PMI: Psoas muscle index, SMD: Skeletal muscle index, ASCT: Autologous stem cell transplantation, ECOG: Eastern Cooperative Oncology Group, ISS: International staging system.

Supplementary Material 2: Supplementary Figure 1. Greater CT Morphometric Decline in Patients with MM Undergoing ASCT. Supplementary Figure 1 illustrates the significantly greater declines in CT morphometric body composition parameters in patients with MM undergoing ASCT compared to those who did not. (A) SMI decreased significantly more in ASCT patients compared to non-ASCT patients (p < 0.001). (B) PSMI showed a significantly greater reduction in ASCT patients than in non-ASCT patients (p < 0.001). (C) PMI exhibited the most pronounced decline, with a significantly greater reduction in ASCT patients compared to non-ASCT patients (p < 0.001). (D) SMD declined similarly in both groups, with no significant difference between ASCT and non-ASCT patients (p = 0.22). (E) VAT showed a significantly greater reduction in ASCT patients compared to non-ASCT patients (p < 0.01). Supplementary Figure 2. CT morphometric decline in muscle-associated parameters and visceral fat in Patients with Multiple Myeloma (MM) under 55 years of age. Analysis of patients with MM under 55 years demonstrated significant declines in several morphometric parameters, indicating that the observed changes were predominantly disease-related rather than attributable to aging. SMI, PSMI, PMI, SMD and VAT declined significantly over the disease course. Supplementary Figure 3. Comparison of relative CT morphometric changes between patients aged<55 and ≥55 years. Supplementary Figure 3 shows the percentage change in (A) skeletal muscle index (SMI), (B) paraspinal muscle index (PSMI), (C) psoas muscle index (PMI), (D) skeletal muscle density (SMD), and (E) visceral adipose tissue (VAT) from the first CT (tCT1) to the third CT (tCT3) in patients with MM stratified by age (<55 vs. ≥55 years). While both subgroups exhibited significant declines in all parameters over the disease course, no statistically significant differences were observed between the two age groups (all p = n.s.).

## Data Availability

No datasets were generated or analysed during the current study.

## References

[1] Brigle K, Rogers B. Pathobiology and diagnosis of multiple myeloma. Semin Oncol Nurs. 2017;33(3):225–36. 28688533 10.1016/j.soncn.2017.05.012

[2] Kazandjian D. Multiple myeloma epidemiology and survival: a unique malignancy. Semin Oncol. 2016;43(6):676–81. 28061985 10.1053/j.seminoncol.2016.11.004 PMC5283695

[3] Palumbo A, Anderson K. Multiple myeloma. N Engl J Med. 2011;364(11):1046–60. 21410373 10.1056/NEJMra1011442

[4] Facon T, Leleu X, Manier S. How i treat multiple myeloma in geriatric patients. Blood. 2024;143(3):224–32. 36693134 10.1182/blood.2022017635 PMC10808246

[5] Diamond E, Lahoud OB, Landau H. Managing multiple myeloma in elderly patients. Leuk Lymphoma. 2018;59(6):1300–11. 28847191 10.1080/10428194.2017.1365859 PMC7494002

[6] Zweegman S, Engelhardt M, Larocca A. Elderly patients with multiple myeloma: towards a frailty approach? Curr Opin Oncol. 2017;29(5):315–21. 28763310 10.1097/CCO.0000000000000395

[7] Antoine-Pepeljugoski C, Braunstein MJ. Management of newly diagnosed elderly multiple myeloma patients. Curr Oncol Rep. 2019;21(7):64. 31127403 10.1007/s11912-019-0804-4

[8] Willan J, et al. Multiple myeloma in the very elderly patient: challenges and solutions. Clin Interv Aging. 2016;11:423–35. 27143866 10.2147/CIA.S89465 PMC4839967

[9] Johnson TM. Multiple myeloma treatment and management in the elderly. Consult Pharm. 2014;29(7):434–8, 440-4, 446–51. 25203105 10.4140/TCP.n.2014.434

[10] Auner HW, Garderet L, Kröger N. Autologous haematopoietic cell transplantation in elderly patients with multiple myeloma. Br J Haematol. 2015;171(4):453–62. 26213240 10.1111/bjh.13608

[11] Suzuki K, et al. Tandem autologous stem cell transplantation in elderly patients with myeloma: a multicenter retrospective analysis. Eur J Haematol. 2023;110(4):444–54. 36597575 10.1111/ejh.13922

[12] Ozaki S, Shimizu K. Autologous stem cell transplantation in elderly patients with multiple myeloma: past, present, and future. Biomed Res Int. 2014;2014:394792. 24719860 10.1155/2014/394792 PMC3956410

[13] Dent E, et al. Malnutrition in older adults. Lancet. 2023;401(10380):951–66. 36716756 10.1016/S0140-6736(22)02612-5

[14] Kim DH, Rockwood K. Frailty in older adults. N Engl J Med. 2024;391(6):538–48. 39115063 10.1056/NEJMra2301292 PMC11634188

[15] Falk Erhag H, et al. The association between the clinical frailty scale and adverse health outcomes in older adults in acute clinical settings - a systematic review of the literature. Clin Interv Aging. 2023;18:249–61. 36843633 10.2147/CIA.S388160 PMC9946013

[16] Mian H, et al. The prevalence and outcomes of frail older adults in clinical trials in multiple myeloma: a systematic review. Blood Cancer J. 2023;13(1):6. 36599867 10.1038/s41408-022-00779-2 PMC9813365

[17] Brown JC, Harhay MO, Harhay MN. Sarcopenia and mortality among a population-based sample of community-dwelling older adults. J Cachexia Sarcopenia Muscle. 2016;7(3):290–8. 27239410 10.1002/jcsm.12073 PMC4864252

[18] Allison DB, et al. Weight loss increases and fat loss decreases all-cause mortality rate: results from two independent cohort studies. Int J Obes Relat Metab Disord. 1999;23(6):603–11. 10411233 10.1038/sj.ijo.0800875

[19] Hiltunen K, et al. Relationship between Fried’s frailty phenotype and oral frailty in long-term care residents. Age Ageing. 2021;50(6):2133–9. 34473831 10.1093/ageing/afab177 PMC8581380

[20] Gahagan A, et al. Evaluating concordance between International Myeloma Working Group (IMWG) frailty score and simplified frailty scale among older adults with multiple myeloma. J Geriatr Oncol. 2024;15(8):102051. 39241344 10.1016/j.jgo.2024.102051

[21] Chowdhury MM, et al. Morphometric assessment as a predictor of outcome in older vascular surgery patients. Ann Vasc Surg. 2018;47:90–7. 28887259 10.1016/j.avsg.2017.08.002

[22] Chianca V, et al. Sarcopenia: imaging assessment and clinical application. Abdom Radiol (NY). 2022;47(9):3205–16. 34687326 10.1007/s00261-021-03294-3 PMC8536908

[23] Laur O, et al. Computed tomography-based body composition profile as a screening tool for geriatric frailty detection. Skeletal Radiol. 2022;51(7):1371–80. 34862921 10.1007/s00256-021-03951-0 PMC8642750

[24] Matsui R, et al. Association of visceral adipose tissue with postoperative outcome in upper gastrointestinal cancer: a systematic review and meta-analysis. Am J Clin Nutr. 2022;116(6):1540–52. 36166841 10.1093/ajcn/nqac273

[25] Liu Z, et al. Deep learning-based radiomics allows for a more accurate assessment of sarcopenia as a prognostic factor in hepatocellular carcinoma. J Zhejiang Univ Sci B. 2024;25(1):83–90. 38163668 10.1631/jzus.B2300363 PMC10758209

[26] Sabel MS, et al. Analytic morphometric assessment of patients undergoing colectomy for colon cancer. J Surg Oncol. 2013;108(3):169–75. 23846976 10.1002/jso.23366

[27] Unamuno X, et al. Adipokine dysregulation and adipose tissue inflammation in human obesity. Eur J Clin Invest. 2018;48(9):e12997. 29995306 10.1111/eci.12997

[28] Zakaria HM, et al. Morphometrics predicts overall survival in patients with multiple myeloma spine metastasis: a retrospective cohort study. Surg Neurol Int. 2018;9:172. 30210905 10.4103/sni.sni_383_17 PMC6122282

[29] Nandakumar B, et al. Sarcopenia identified by computed tomography imaging using a deep learning-based segmentation approach impacts survival in patients with newly diagnosed multiple myeloma. Cancer. 2023;129(3):385–92. 36413412 10.1002/cncr.34545 PMC9822865

[30] Surov A, et al. CT-defined muscle density as a prognostic factor in multiple myeloma undergoing autologous stem cell therapy: a retrospective single center study. J Cancer Res Clin Oncol. 2024;150(11):499. 39546043 10.1007/s00432-024-06009-5 PMC11567988

[31] Kumar S, et al. International Myeloma Working Group consensus criteria for response and minimal residual disease assessment in multiple myeloma. Lancet Oncol. 2016;17(8):e328–46. 27511158 10.1016/S1470-2045(16)30206-6

[32] Leitlinienprogramm Onkologie (Deutsche Krebs- gesellschaft, Deutsche Krebshilfe, AWMF Diag- nostik, Therapie und Nachsorge für Patienten mit monoklonaler Gammopathie unklarer Signifikanz (MGUS) oder Multiplen Myelom, Langversi- on 1.01 (Konsultationsfassung, 2021, AWMF Registernummer: 018/035OL. https://www.Leitlinienprogramm-Onkologie.De/Leitlinien/Multiples-Myelom/. 2021.

[33] Albano D, et al. Imaging of sarcopenia: old evidence and new insights. Eur Radiol. 2020;30(4):2199–208. 31834509 10.1007/s00330-019-06573-2

[34] Palumbo A, et al. Revised international staging system for multiple myeloma: a report from International Myeloma Working Group. J Clin Oncol. 2015;33(26):2863–9. 26240224 10.1200/JCO.2015.61.2267 PMC4846284

[35] Cole CL, et al. The role of systemic inflammation in cancer-associated muscle wasting and rationale for exercise as a therapeutic intervention. JCSM Clinical Reports. 2018. 10.17987/jcsm-cr.v3i2.65. 31134216 PMC6534125

[36] Anderson KC, Lust JA. Role of cytokines in multiple myeloma. Semin Hematol. 1999;36(1 Suppl 3):14–20. 9989484

[37] Agca S, Kir S. The role of interleukin-6 family cytokines in cancer cachexia. FEBS J. 2024;291(18):4009–23. 38975832 10.1111/febs.17224

[38] Hirano T. IL-6 in inflammation, autoimmunity and cancer. Int Immunol. 2021;33(3):127–48. 33337480 10.1093/intimm/dxaa078 PMC7799025

[39] Mielnik M, et al. The clinical relevance of selected cytokines in newly diagnosed multiple myeloma patients. Biomedicines. 2023. 10.3390/biomedicines11113012. 38002012 10.3390/biomedicines11113012 PMC10669681

[40] Masi T, Patel BM. Altered glucose metabolism and insulin resistance in cancer-induced cachexia: a sweet poison. Pharmacol Rep. 2021;73(1):17–30. 33141425 10.1007/s43440-020-00179-y

[41] Liu JP, et al. Impact of myosteatosis on prognosis in multiple myeloma patients: a subgroup analysis of 182 cases and development of a nomogram. J Bone Oncol. 2025;51:100670. 40162121 10.1016/j.jbo.2025.100670 PMC11952022

[42] Lee K, et al. Telehealth exercise to improve physical function and frailty in patients with multiple myeloma treated with autologous hematopoietic stem cell transplantation (TIPS): protocol of a randomized controlled trial. Trials. 2022;23(1):921. 36329525 10.1186/s13063-022-06848-y PMC9633031

[43] Normann AJ, et al. Prehabilitation exercise training to target improved muscle strength in pretransplant patients diagnosed with multiple myeloma: protocol for a pilot randomized controlled trial. JMIR Res Protoc. 2024;13:e64905. 39701583 10.2196/64905 PMC11695955

