# Supplementary material for: Sarcopenia and fat loss from serial CT predict survival in multiple myeloma patients undergoing stem cell transplantation
Source: World J Surg Oncol. 2025 Sep 17;23:336. doi: 10.1186/s12957-025-04007-6 (PMC12442264; doi:10.1186/s12957-025-04007-6)
Supplement: Supplementary file 1 — Supplementary Material 1: Supplementary Table 1. CT-morphometric and clinical parameters over the disease course. Supplementary Table 1 shows the values and significance levels of the Uni- and multivariable Cox regression models for overall survival. Abbreviations: SMI: Skeletal muscle index, VAT: Visceral adipose tissue, PSMI: Paraspinal muscle index, PMI: Psoas muscle index, SMD: Skeletal muscle index, ASCT: Autologous stem cell transplantation, ECOG: Eastern Cooperative Oncology Group, ISS: International staging system. [file 12957_2025_4007_MOESM1_ESM.docx]

**Supplementary Table 1 - CT-morphometric and clinical parameters over the disease course**

|  | **tCT1 (Mean/SD)** | **tCT2 (Mean/SD)** | **tCT3 (Mean/SD)** |
| --- | --- | --- | --- |
| **Skeletal Muscle Index (SMI) [cm^2^/m^2^]** | **43.5 (7.4)** | **35.8 (7.6)** | **32.9 (6.9)** |
| **Male** | **48.6 (7.1)** | **40.6 (6.0)** | **38.4 (8.1)** |
| **Female** | **36.6 (7.8)** | **30.5 (8.0)** | **26.9 (4.7)** |
| **Paraspinal Muscle Index (PSMI) [cm^2^/m^2^]** | **16.1 (0.7)** | **14.9 (0.9)** | **13.3 (0.7)** |
| **Male** | **16.7 (0.5)** | **14.9 (0.8)** | **12.4 (0.7)** |
| **Female** | **16.9 (0.9)** | **14.1 (0.6)** | **13.9 (0.8)** |
| **Psoas Muscle Index (PMI) [cm^2^/m^2^]** | **2.7 (0.4)** | **1.9 (0.3)** | **1.5 (0.7)** |
| **Male** | **2.8 (0.6)** | **2.4 (0.4)** | **1.8 (0.7)** |
| **Female** | **2.5 (0.3)** | **1.6 (0.4)** | **1.3 (0.7)** |
| **Skeletal Muscle Density (SMD) [cm^2^/m^2^]** | **39.8 (7.0)** | **34.0 (8.2)** | **30.3 (7.1)** |
| **Male** | **40.1 (8.2)** | **34.8 (8.0)** | **31.9 (10.0)** |
| **Female** | **38.7 (9.1)** | **34.3 (10.1)** | **28.7 (8.2)** |
| **Visceral Adipose Tissue (VAT) [cm^2^/m^2^]** | **89.0 (9.4)** | **76.9 (9.6)** | **63.4 (8.0)** |
| **Male** | **115.9 (9.1)** | **100.0 (9.1)** | **84.9 (9.7)** |
| **Female** | **63.5 (7.1)** | **52.9 (9.1)** | **42.1 (7.7)** |
| **ECOG** | **1 (1)** | **1 (4)** | **2 (3)** |
| **VAS** | **2 (9)** | **4 (9)** | **6 (9)** |
| **WHO** | **1 (3)** | **1 (3)** | **3 (3)** |
