# Supplementary figures and images for: Sarcopenia and fat loss from serial CT predict survival in multiple myeloma patients undergoing stem cell transplantation
Source: World J Surg Oncol. 2025 Sep 17;23:336. doi: 10.1186/s12957-025-04007-6 (PMC12442264; doi:10.1186/s12957-025-04007-6)

**Supplementary Figure 1**

A

B

C

D

E


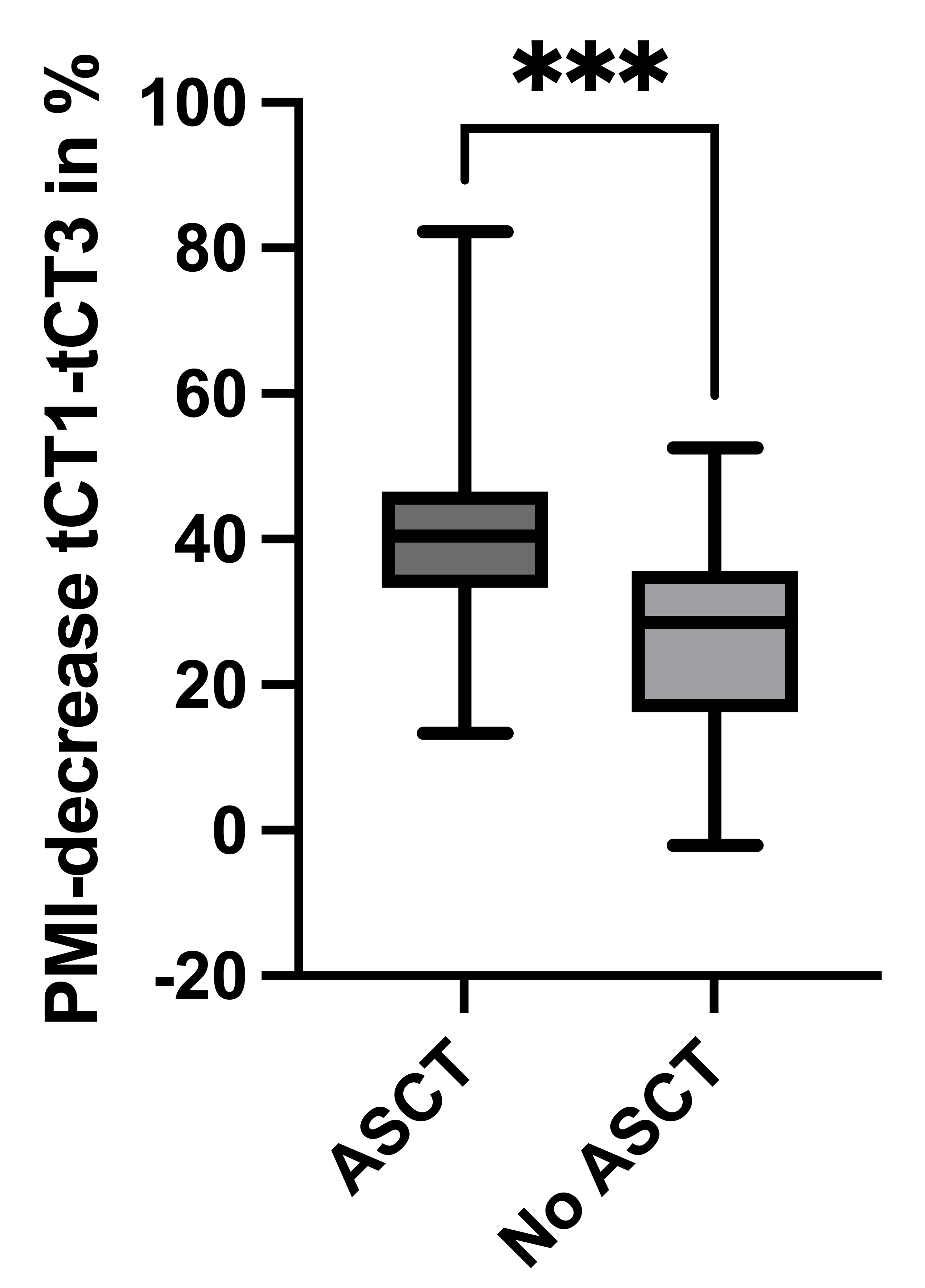

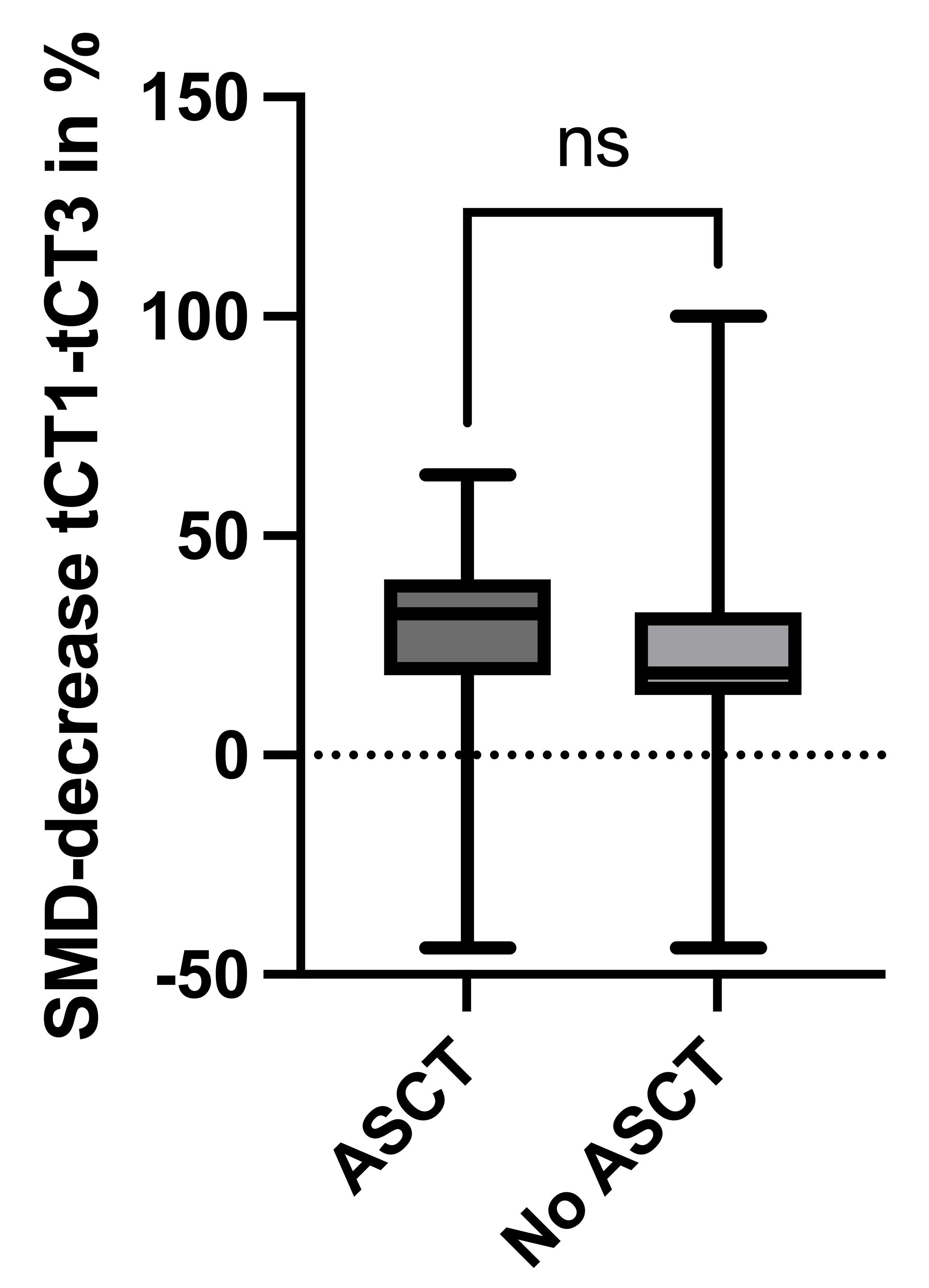

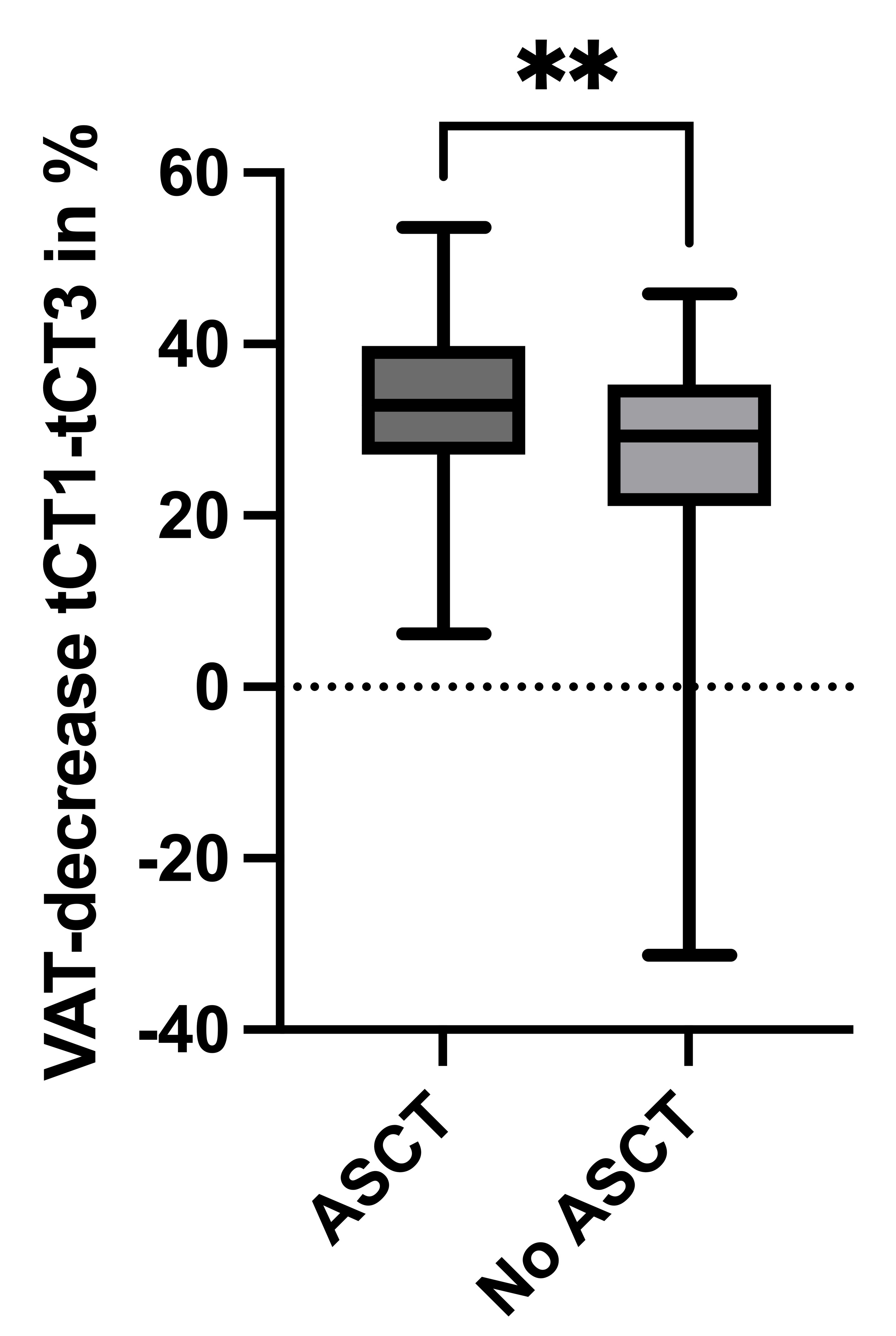

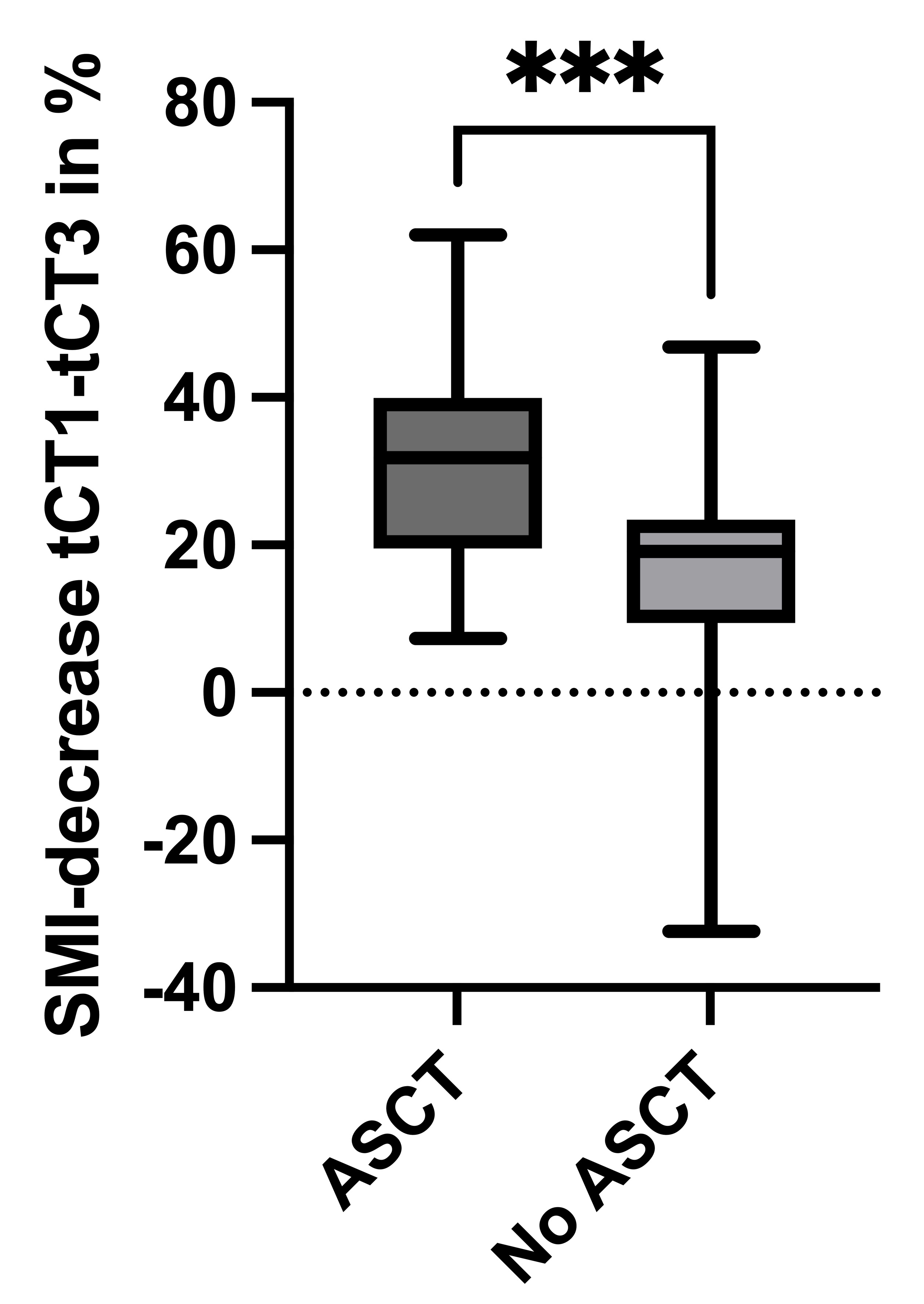

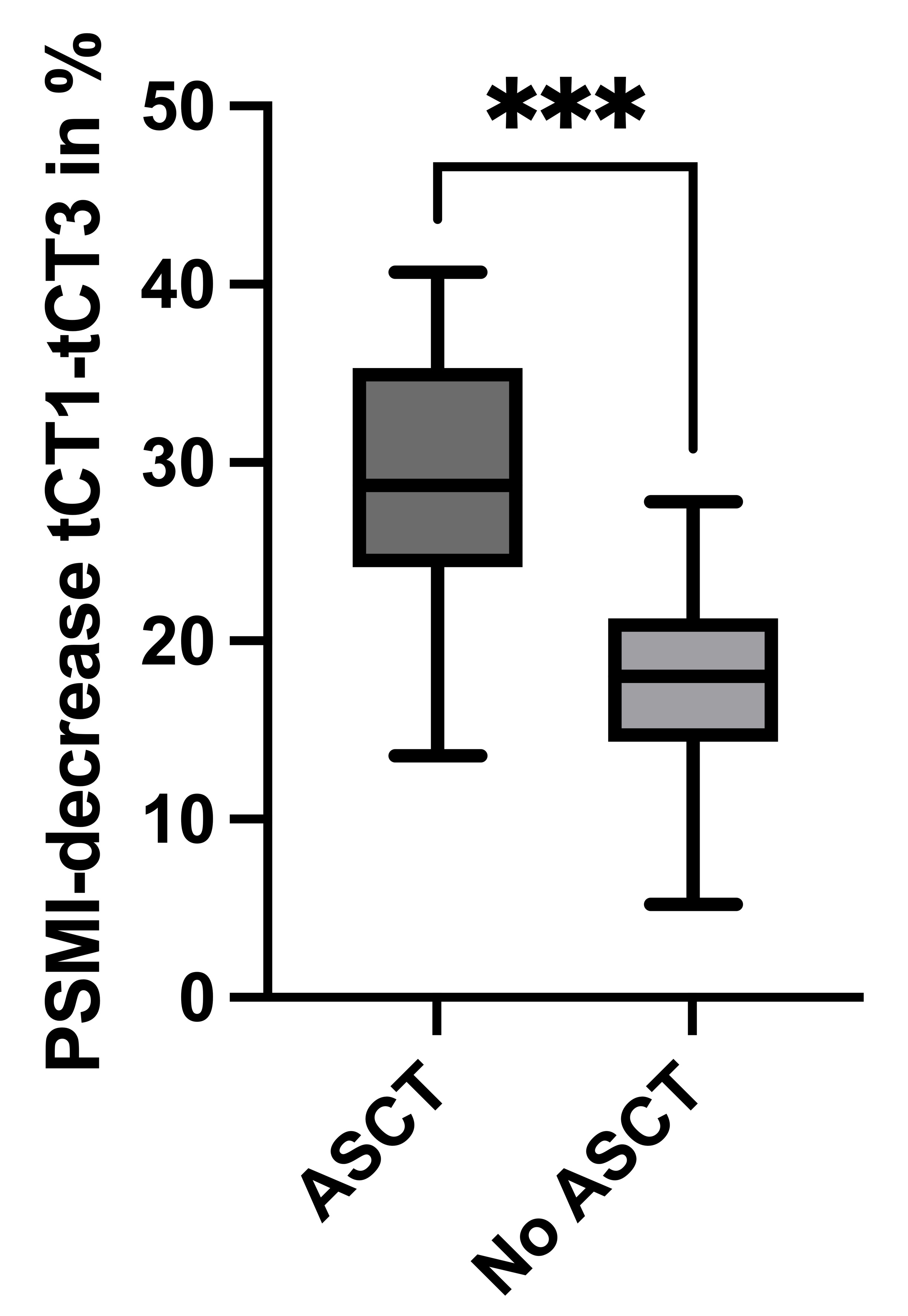


**Supplementary Figure 2**

A

B

C

D

E


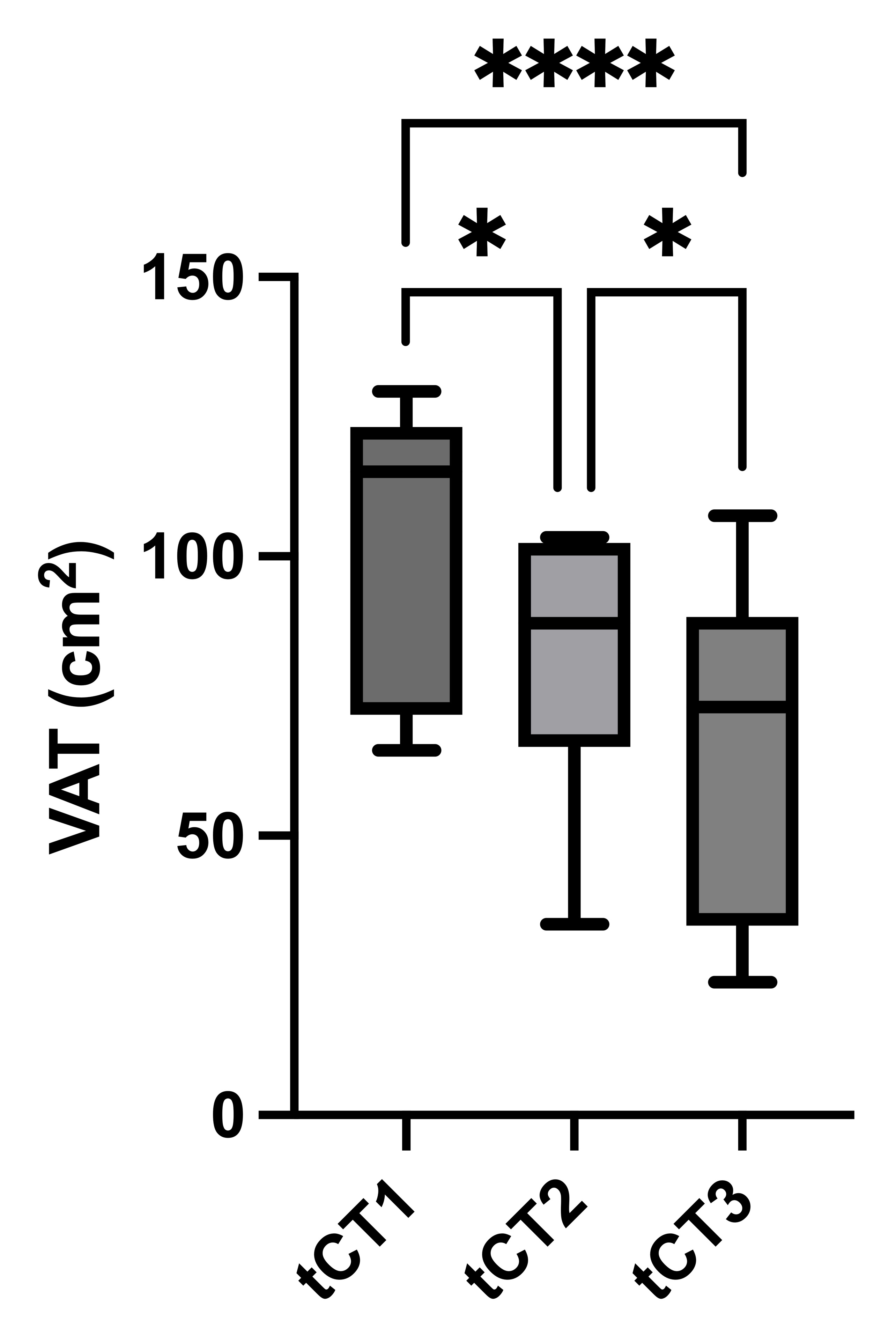

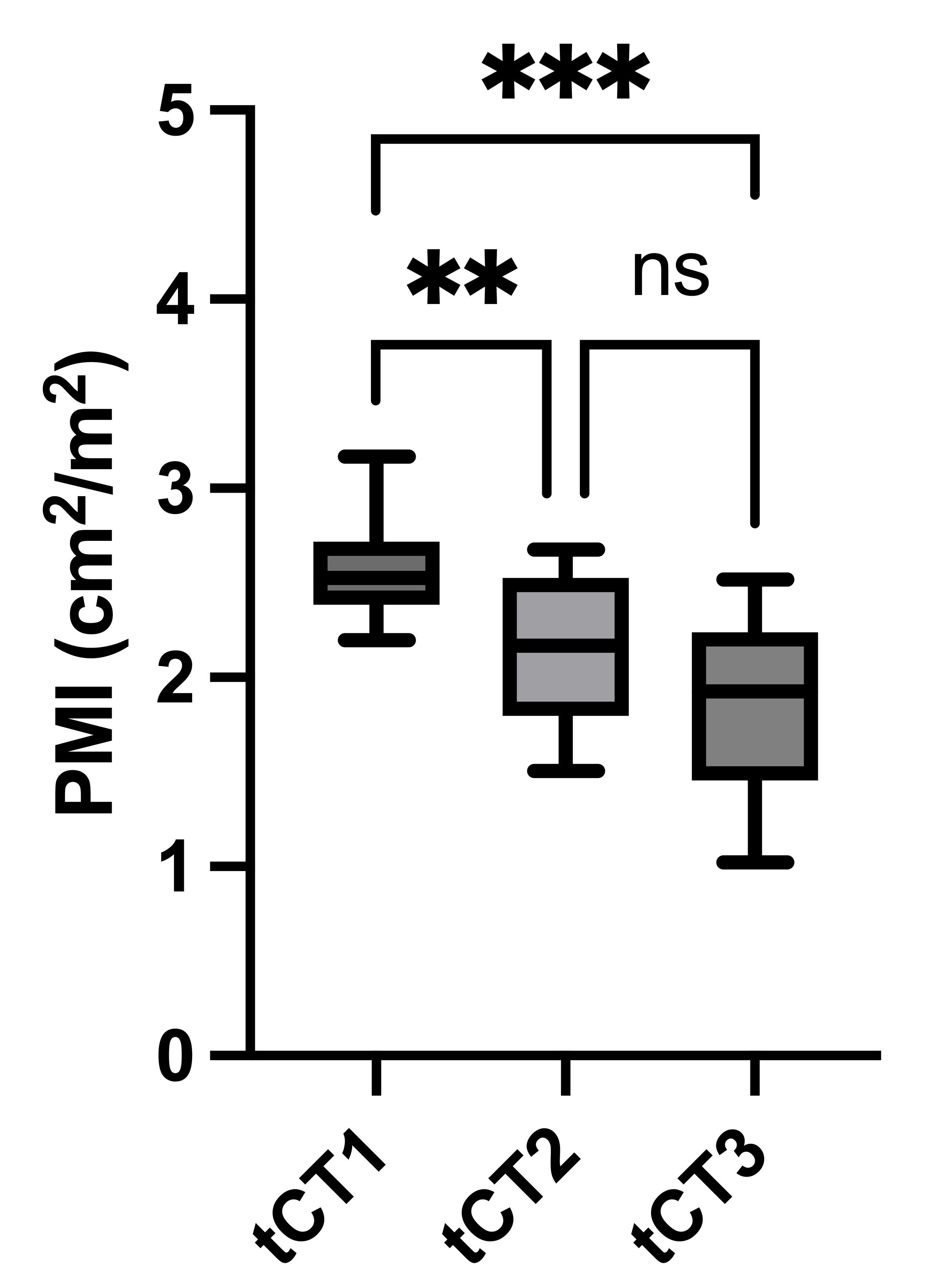

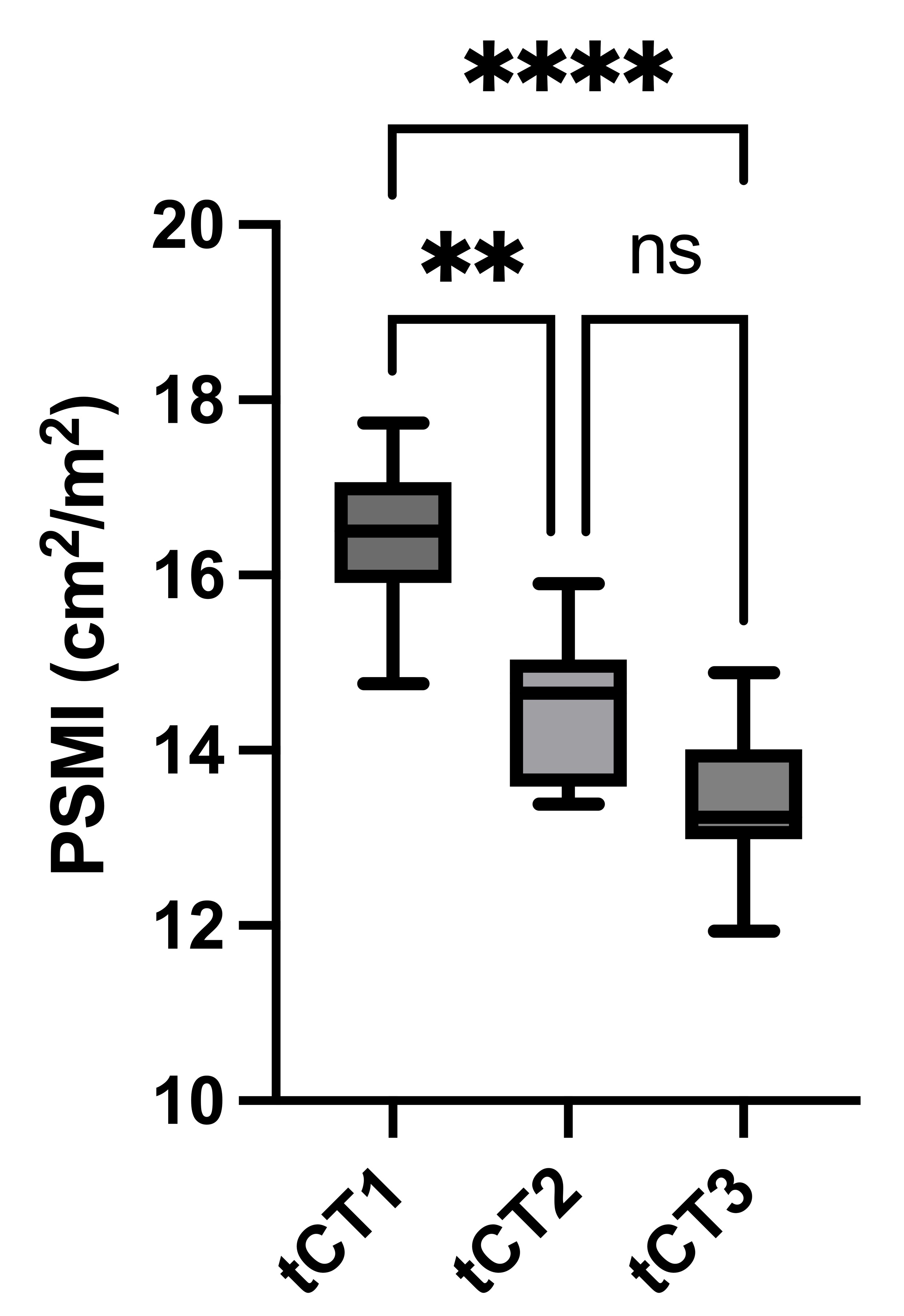

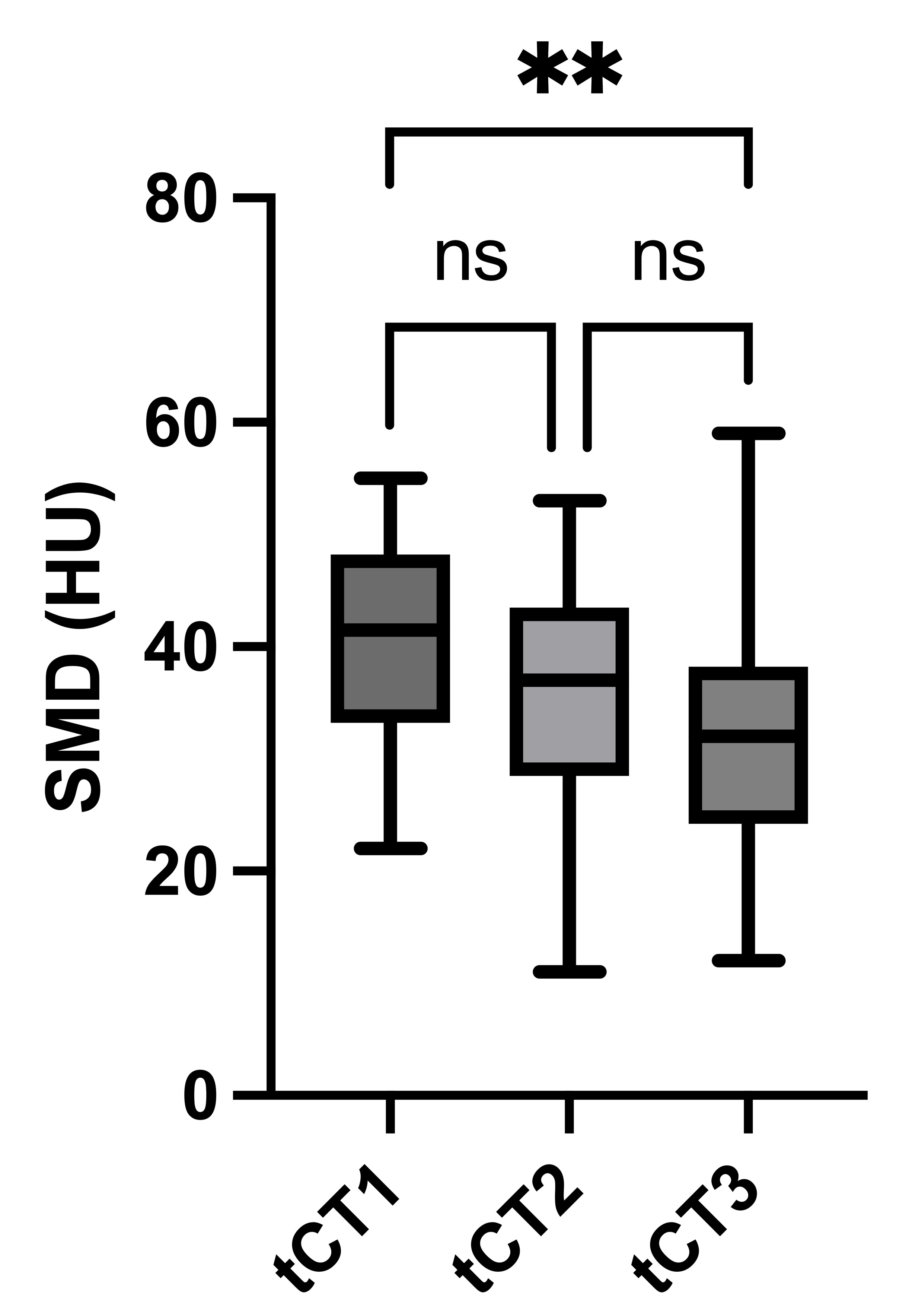

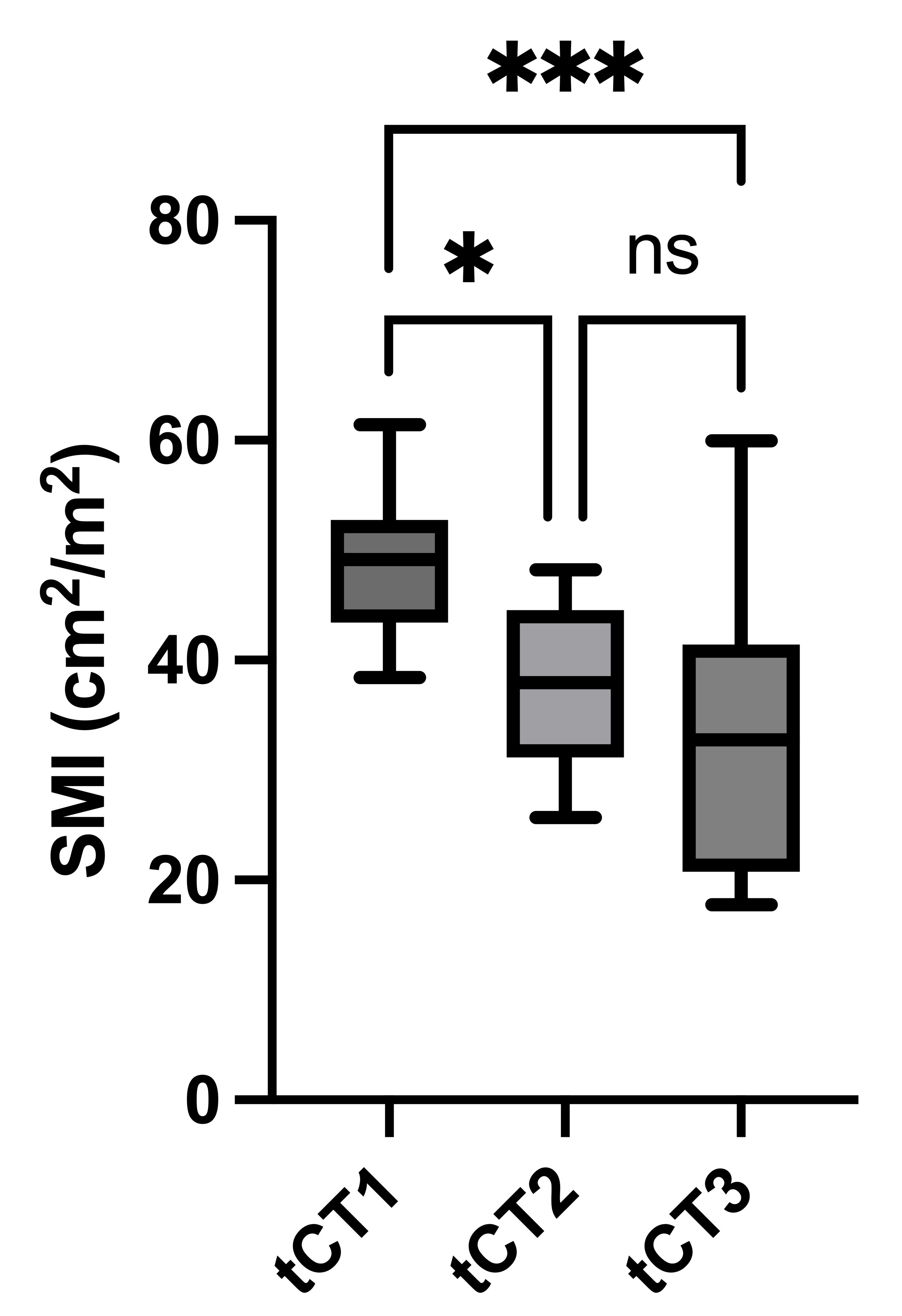


**Supplementary Figure 3**

A

B

C

D

E


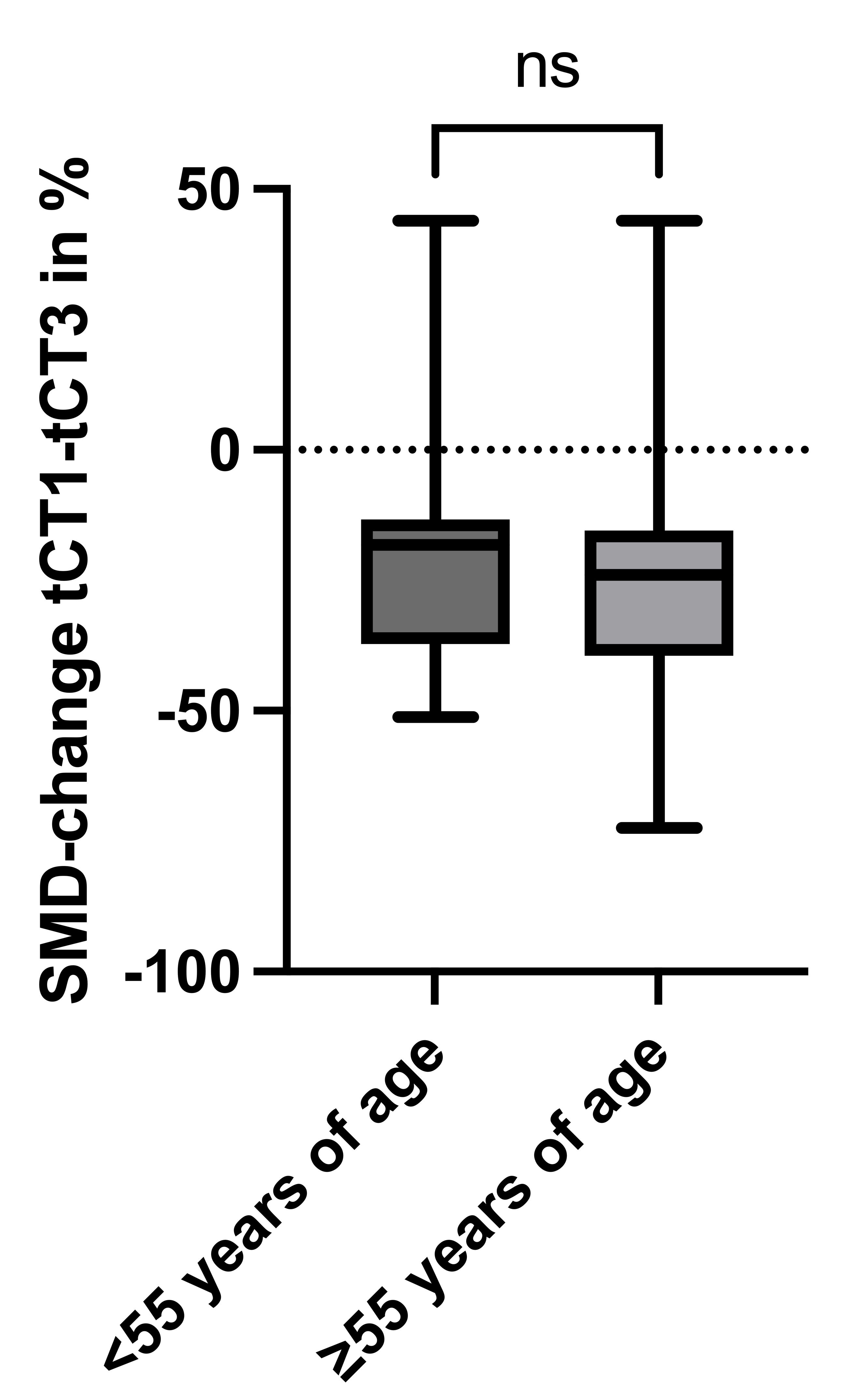

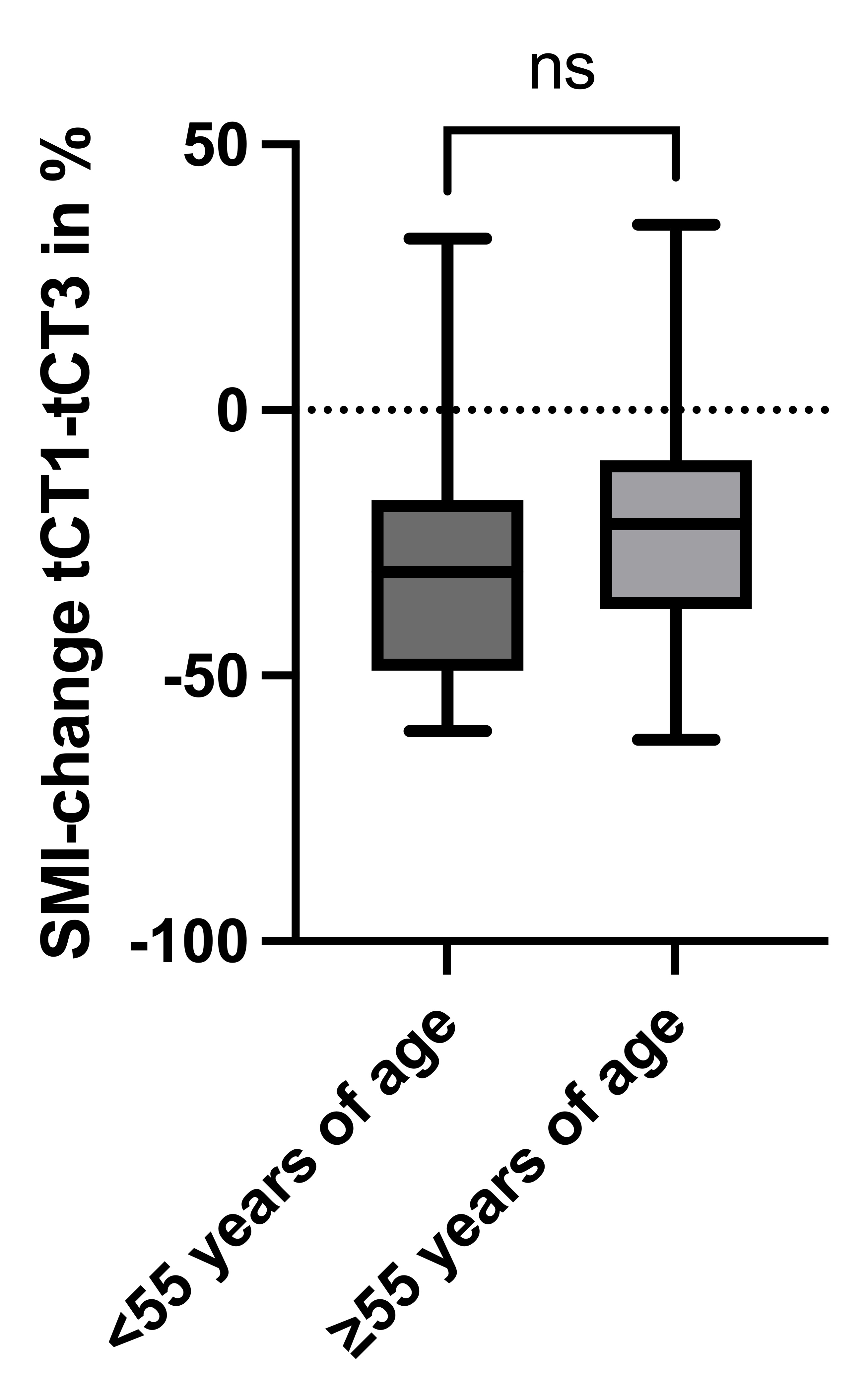

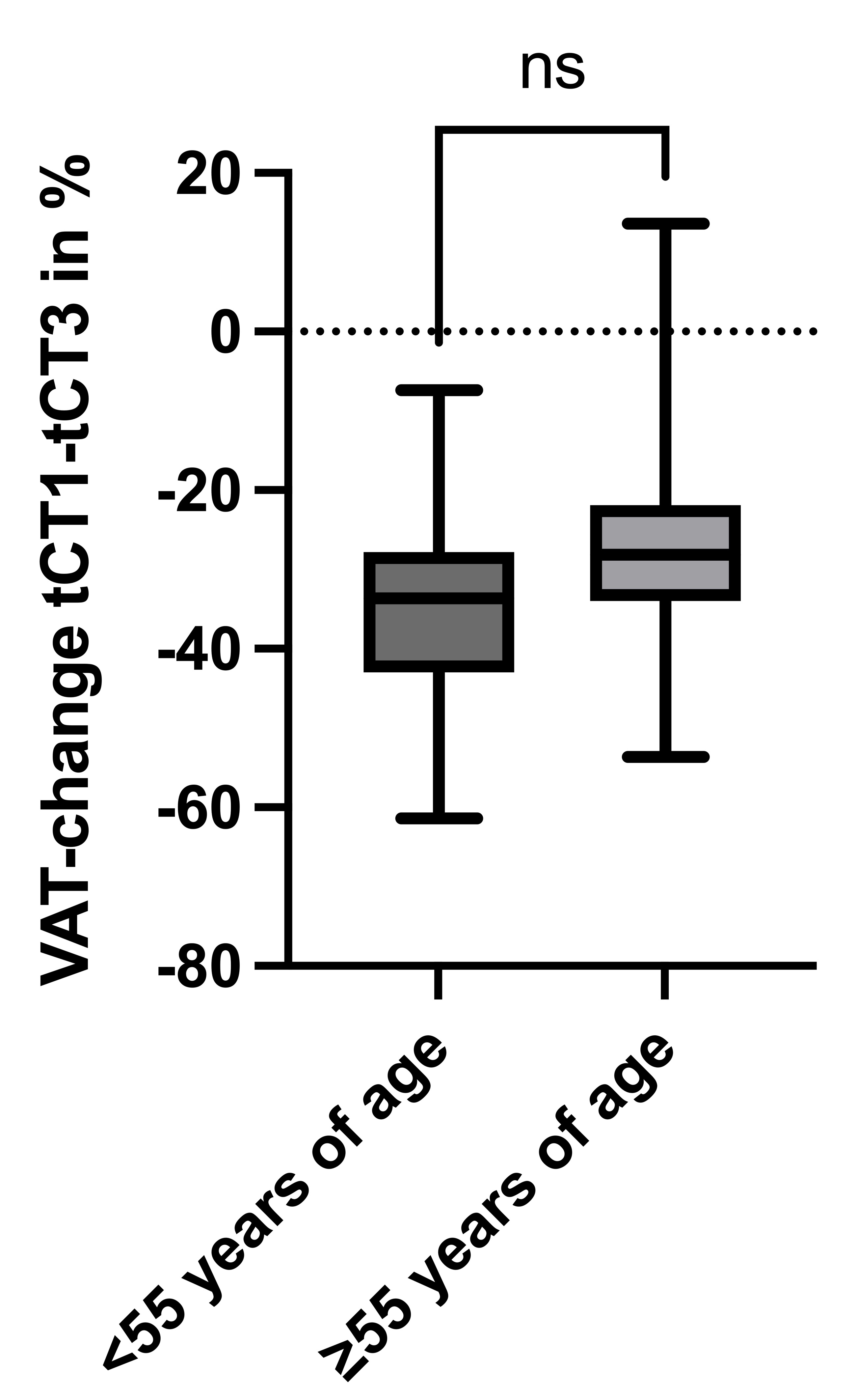

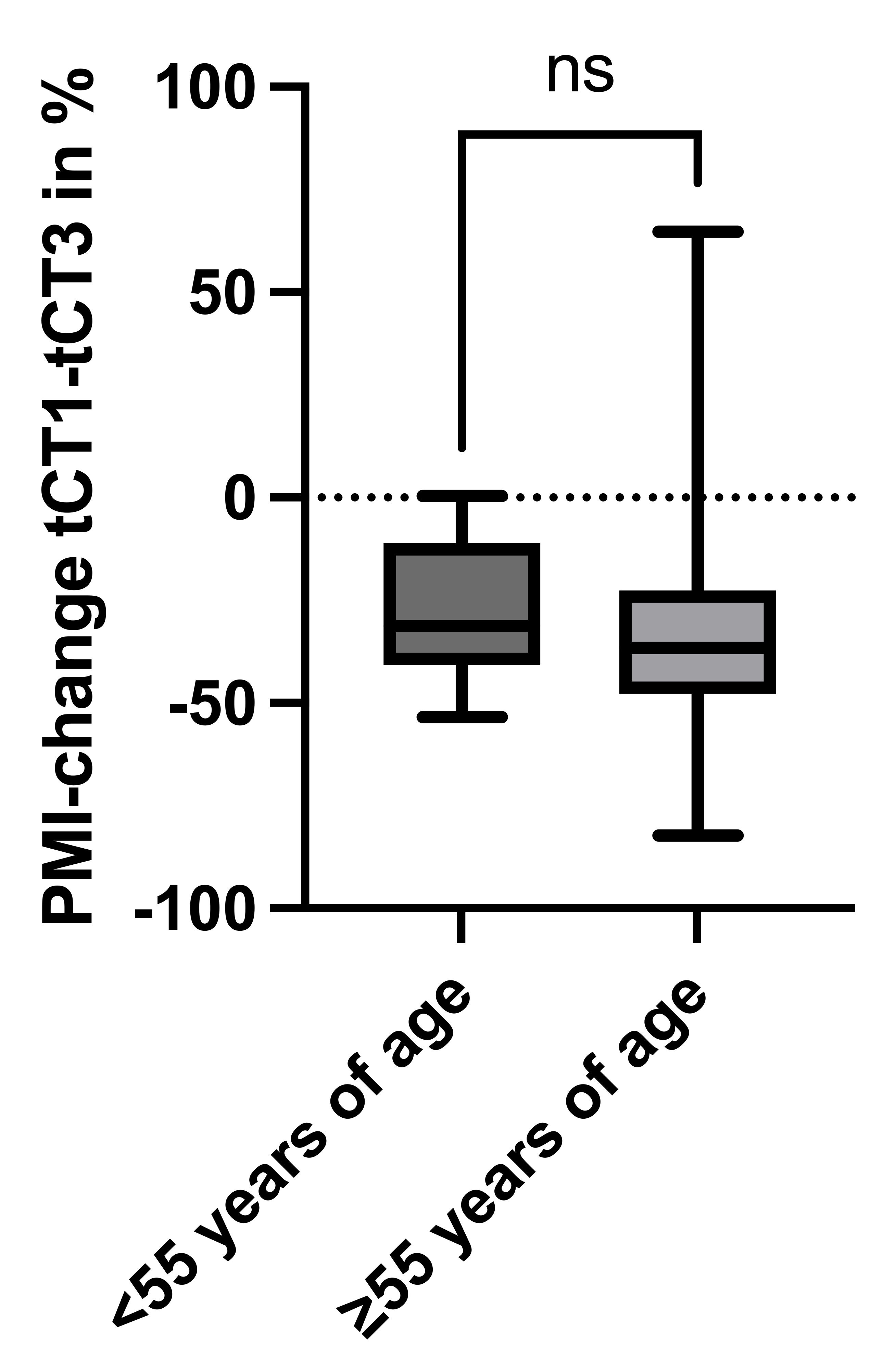

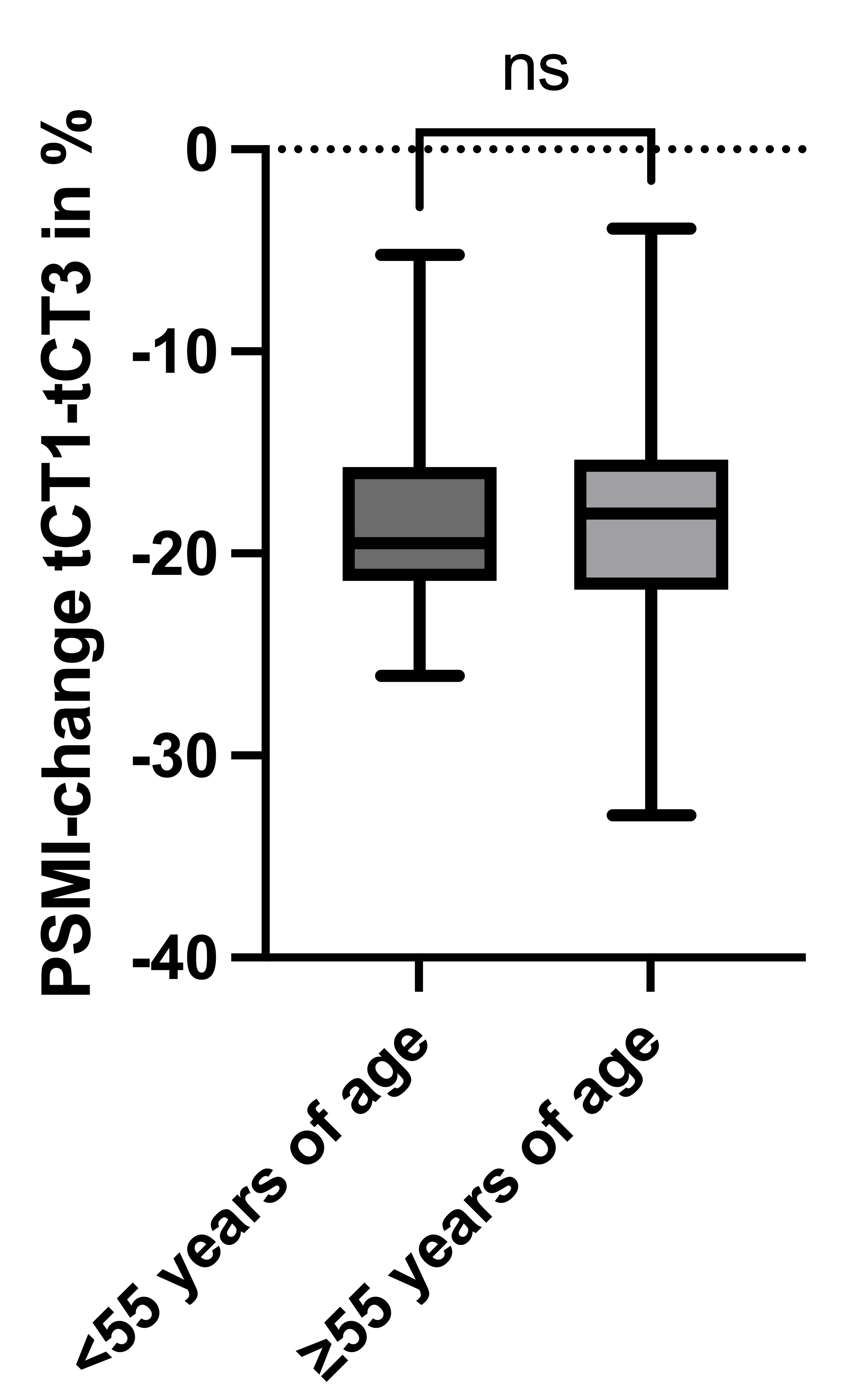

Supplement: Supplementary file 2 — Supplementary Material 2: Supplementary Figure 1. Greater CT Morphometric Decline in Patients with MM Undergoing ASCT. Supplementary Figure 1 illustrates the significantly greater declines in CT morphometric body composition parameters in patients with MM undergoing ASCT compared to those who did not. (A) SMI decreased significantly more in ASCT patients compared to non-ASCT patients (p < 0.001). (B) PSMI showed a significantly greater reduction in ASCT patients than in non-ASCT patients (p < 0.001). (C) PMI exhibited the most pronounced decline, with a significantly greater reduction in ASCT patients compared to non-ASCT patients (p < 0.001). (D) SMD declined similarly in both groups, with no significant difference between ASCT and non-ASCT patients (p = 0.22). (E) VAT showed a significantly greater reduction in ASCT patients compared to non-ASCT patients (p < 0.01). Supplementary Figure 2. CT morphometric decline in muscle-associated parameters and visceral fat in Patients with Multiple Myeloma (MM) under 55 years of age. Analysis of patients with MM under 55 years demonstrated significant declines in several morphometric parameters, indicating that the observed changes were predominantly disease-related rather than attributable to aging. SMI, PSMI, PMI, SMD and VAT declined significantly over the disease course. Supplementary Figure 3. Comparison of relative CT morphometric changes between patients aged<55 and ≥55 years. Supplementary Figure 3 shows the percentage change in (A) skeletal muscle index (SMI), (B) paraspinal muscle index (PSMI), (C) psoas muscle index (PMI), (D) skeletal muscle density (SMD), and (E) visceral adipose tissue (VAT) from the first CT (tCT1) to the third CT (tCT3) in patients with MM stratified by age (<55 vs. ≥55 years). While both subgroups exhibited significant declines in all parameters over the disease course, no statistically significant differences were observed between the two age groups (all p = n.s.). [file 12957_2025_4007_MOESM2_ESM.docx]
